# Reconstruction of Acoustic Echoes and Their Effect on Brillouin Oscillations in Nanoscale Characterization of Supported Thin Films by Ultrafast Photoacoustics

**DOI:** 10.3390/nano16201307

**Published:** 2026-10-16

**Authors:** Sergey A. Romashevskiy

**Affiliations:** Joint Institute for High Temperatures of the Russian Academy of Sciences, Izhorskaya st. 13 Bd.2, 125412 Moscow, Russia; sa.romashevskiy@gmail.com

**Keywords:** nanoscale film, thin-film metal–dielectric structure, femtosecond laser, transient reflectivity, picosecond acoustics, coherent acoustic phonons, time-domain Brillouin scattering, Brillouin oscillations, amplitude-phase modulation

## Abstract

The paper presents an experimental and theoretical analysis of the effects accompanying the non-contact, non-destructive nanoscale characterization of thin-film metal–dielectric structures through the substrate, using high-amplitude picosecond acoustic pulses generated by an ultrafast laser. Time-resolved reflectivity changes Δ*R*(*t*)/*R*_0_ at 793 nm are measured in the picosecond time domain using an all-optical pump-probe setup with balanced, phase-sensitive detection tuned to 0.5 kHz. For a glass-supported nanoscale nickel film, the measurements at the glass–film interface are carried out at absorbed laser fluences ranging from 1.7 to 10.3 mJ/cm^2^, generating peak pressures of up to several gigapascals. The Δ*R*(*t*)/*R*_0_ signal is dominated by the resulting Brillouin oscillations at a frequency of *f*_BO_ = 1/*T*_BO_ = 21.5 ± 0.1 GHz within the substrate, which undergo amplitude-phase modulation due to the superposition of the individual Brillouin oscillations from the acoustic pulses leaking into the substrate after reverberating in the film. Through post-processing, the obscured Δ*R*(*t*)/*R*_0_ signal of the acoustic pulses reverberating in the film at *f*_echo_ = 1/*T*_echo_ = 39.1 ± 0.2 GHz is reconstructed, showing excellent agreement with the reference signal directly measured at the air–film interface. It is shown that, unlike sufficiently thick submicron films, which exhibit no amplitude-phase modulation by reverberating acoustic pulses during the first several hundred picoseconds (*T*_echo_ >> *T*_BO_), nanoscale films—where *T*_echo_ ≈ *T*_BO_/2—cause a significant reduction in the resulting amplitude of the Brillouin oscillations in the substrate, which reaches its minimum due to destructive interference.

## 1. Introduction

Nanoscale thin-film materials and interfaces, supported by advanced fabrication technologies, comprehensive characterization, and diagnostic methodologies, form the foundation of modern high-tech industries and remain a critical driver for innovation [1,2,3,4,5,6,7]. Continuous progress in film deposition methods is essential for the scaling of basic electronic components and the performance enhancement of integrated circuits, photovoltaics, micro- and nanoelectronic devices [8,9,10]. Furthermore, it facilitates the development and implementation of non-destructive, contactless, and remote monitoring techniques that provide nanoscale resolution [11,12,13,14].

Probing with ultrashort acoustic pulses induced by ultrashort light pulses—referred to as picosecond acoustics and time-domain Brillouin scattering (or picosecond interferometry) [15,16,17,18,19]—has emerged as a promising method for investigating both transparent condensed media and absorbing solid-state materials, generally with submicron lateral and nanoscale longitudinal spatial resolutions [20,21,22,23,24,25,26,27]. However, a nanoscale lateral resolution of up to 270 nm can be achieved by coupling picosecond acoustics with atomic force microscopy, or focusing the acoustic beam down to 250 nm using planar Fresnel zone plate and concave lenses [28,29]. Picosecond acoustics relies on the generation of high-frequency coherent acoustic phonons in the gigahertz–terahertz frequency range, driven by instantaneous heating and thermoelastic expansion of the material’s irradiated volume following the absorption of an exciting (pump) laser pulse [30,31]. Propagating acoustic pulses induce a local relative change in density Δ*ρ*/*ρ*_0_, which modifies the material’s complex permittivity [17]. The detection of these propagating acoustic pulses is typically accomplished by monitoring the transient changes in the intensity of the reflected Δ*R*(*t*)/*R*_0_ or/and transmitted Δ*T*(*t*)/*T*_0_ probe pulses using an all-optical pump-probe setup coupled with balanced and lock-in detection methods. Depending on parameters such as pump fluence, probe wavelength, and the material’s optical response at a specific probing wavelength, the experimentally measured Δ*R*(*t*)/*R*_0_ and Δ*T*(*t*)/*T*_0_ changes span several orders of magnitude, typically from 10^−7^ to 10^−3^ [16,18,32,33].

In absorbing materials (e.g., metals), the optical detection of propagating acoustic pulses is limited to the optical penetration depth [16,31], meaning they are detectable only upon the acoustic pulse reaching an interface. The acoustic mismatch and diffuse mismatch models are generally used to describe phonon transmission across material interfaces [6,34,35,36,37]. In contrast, in optically transparent materials (solid dielectrics [38], liquids [39,40], and semiconductors [41,42,43]), the detection of acoustic pulses occurs during their propagation within the material as a result of the inelastic scattering of the probing laser pulse by longitudinal acoustic waves [15,17,23]. The scattered laser pulse acquires a Brillouin frequency shift in the gigahertz range, which arises from the dynamic reflection of the laser pulse by a moving acoustic pulse, consisting of compression and tension pressure regions; this is often described in terms of the Doppler effect [44,45]. Experimentally, the propagating acoustic pulses are detected via optical heterodyning: the extremely weak optical signal scattered by the moving acoustic pulse interferes on a photodetector with a strong optical signal reflected from the stationary interfaces of the sample. As a result, high-frequency beating of transient reflectivity or transmissivity changes—Brillouin oscillations (BO)—is produced.

Studies of thermal and acoustic resistance at the metal–dielectric interface—especially in the case of film thicknesses commensurate with the mean free path of carriers (electrons and phonons), where carrier scattering by surfaces dominates [46]—are of considerable interest. The investigation of thin-film materials at extreme temperatures and pressures [21,47,48] plays an important role in assessing the ultimate potential of the devices being developed.

It is worth noting that picosecond acoustics and time-domain Brillouin scattering also correspond to the current trend of transition from *ex situ* to *in situ* monitoring, allowing for the real-time adjustment of deposition parameters and the correction of object alignment [49]. Significant progress has been achieved in both research areas. In picosecond acoustics, advances include the measurement of film thickness and the speed of sound [50,51], the detection of hidden interfaces [49], the assessment of adhesion [52,53], and the investigation of effects stemming from the roughness of optoacoustic transducers and pump pulse repetition rates [54]. In time-domain Brillouin scattering, key achievements involve the detection of nanoscale structural inhomogeneities, including porosity variations [55,56]; the identification of doped layers [57,58]; the mapping of local crystallographic texture [59,60]; and the assessment of local temperature fields [39]. Furthermore, this technique allows for the determination of the attenuation coefficient of propagating high-frequency (>10^9^ Hz) coherent acoustic pulses [17,61], the evaluation of probe pulse spectrum width effects on attenuation [62,63], adhesion assessments [64], and the simultaneous extraction of the refractive index, thickness of transparent layers, and sound velocity [65,66]. Finally, theoretical studies have addressed crucial aspects such as the detection of hidden interfaces located at various angles to the probing laser radiation and the optimization of the ratio between lateral and longitudinal spatial resolutions [67,68].

However, several critically important aspects remain unexplored. These are related to the optoacoustic probing, particularly of nanoscale to microscale thin-film metal–dielectric structures through a transparent substrate [65,69,70,71], including transparent layers of nanoscale thickness [72]. One aspect concerns the reconstruction of acoustic pulses (echoes) reverberating in an underlying nanoscale absorbing (e.g., metal) film, which are otherwise buried in the high-amplitude, quasi-monochromatic Brillouin oscillations within a covering (semi)-transparent substrate. Recovering the time-resolved signal of acoustic echoes provides vital information regarding the film’s thickness, sound velocity, and interfacial quality. Another aspect is closely related to the former, and concerns the effect of reverberating acoustic pulses on the resulting waveform of Brillouin oscillations—specifically their amplitude and phase shift—within the substrate [65]. This is of great significance as the waveform of Brillouin oscillations reflects the local acoustic (sound velocity, elastic and attenuation constants), optical (refractive index), and acousto-optic (photoelastic constants) properties of a material as a coherent acoustic pulse propagates through it [23]. Abrupt changes, phase shifts or frequency variations in the waveform could indicate various local structural inhomogeneities and buried interfaces [23]. Therefore, to unambiguously determine the above-mentioned parameters one must be aware of all the effects caused by the chosen source of laser-induced hypersound itself (e.g., a combination of an optoacoustic transducer film deposited onto the substrate under study). By contrast, the effect of probing photon energy on the amplitude of Brillouin oscillations has been extensively studied in a great number of semiconductors and heterostructures [62,73,74,75], but only a few dielectrics [76,77]. For semiconductors, the highest amplitude was observed for the probing photon energy near the maxima of the derivatives of the complex dielectric function [73,74,75], for example, at the minimum direct optical transition energy of 2.76 eV for GaP [73]. In dielectrics, as shown for a 170 μm thick borosilicate glass coated with a 20 nm Au layer and probed through the substrate, the amplitude of Brillouin oscillations was found to increase with an increase in the probing photon energy from 2.07 to 3.1 eV, while no signal was detected at 1.55 eV [76]. The specific modulation of Brillouin oscillations, which manifests as a stepwise change (increase or decrease) in the Δ*R*/*R*_0_ signal arising from the reflection of acoustic pulses from the free boundary, was found to depend on the probing photon energy (2.75–3.26 eV) and the thickness of the silicon dioxide layer (430–860 nm) [77,78]. In contrast, the suppression of Brillouin oscillations in micron-thick (1.7–4 µm) free-standing diamond films was demonstrated by depositing 10 nm thick Pt films on both sides [79].

This paper experimentally and theoretically covers the two above-stated problems, which are related to (1) the reconstruction of the decaying signal of transient reflectivity changes, caused by the reverberation of acoustic pulses in the film (echoes) and obscured by the high-amplitude Brillouin oscillations within the substrate, and (2) the effect of these acoustic echoes leaking from the film into the substrate on the amplitude-phase modulation of the resulting waveform of Brillouin oscillations. Here, glass-supported nickel thin films are experimentally investigated in the back-pump/back-probe configuration, meaning the pump and probe femtosecond laser pulses are focused through the transparent substrate onto the glass–nickel interface. For a glass-supported nanometer-scale nickel film, the reconstruction of the optical response waveform, caused by acoustic pulses reverberating in the nickel film and buried by strong Brillouin oscillations within the glass substrate, is performed using mathematical post-processing of the experimental measurements of the transient reflectivity change Δ*R*(*t*)/*R*_0_ signal across a maximum achievable range of relatively high non-destructive absorbed pump (exciting) fluences from 1.7 to 10.3 mJ/cm^2^. The reconstructed echo-induced optical response waveform is found to be in agreement with the directly measured echo-induced signal at the air–nickel interface in a front-pump/front-probe configuration. The maximum absorbed fluence of 10.3 mJ/cm^2^ at a repetition rate of 0.5 kHz is determined to be the upper threshold for non-destructive testing, as a further increase in fluence is found to result in the degradation and the destruction of the nanoscale nickel film acting as the optoacoustic transducer. The effect of optoacoustic transducer film thickness on the amplitude and phase of the resulting waveform of Brillouin oscillations within the transparent substrate is investigated experimentally and theoretically for nanoscale and submicroscale nickel films.

## 2. Materials and Methods

A sample consisting of a 150 μm borosilicate glass substrate coated with a nanometer-scale (*d* = 70 nm) polycrystalline nickel film, deposited using magnetron deposition in an Ar atmosphere at 10^−3^ Torr, was used in the experiments. The film thickness was measured using an atomic force microscope (MultiMode NanoScope V, Veeco Instruments Inc., USA) in the tapping mode. Here, the nanoscale nickel film acts both as an optoacoustic transducer for generating high-frequency coherent acoustic phonons and also as a film under study, the acoustic properties of which are to be investigated.

**Figure 1:**
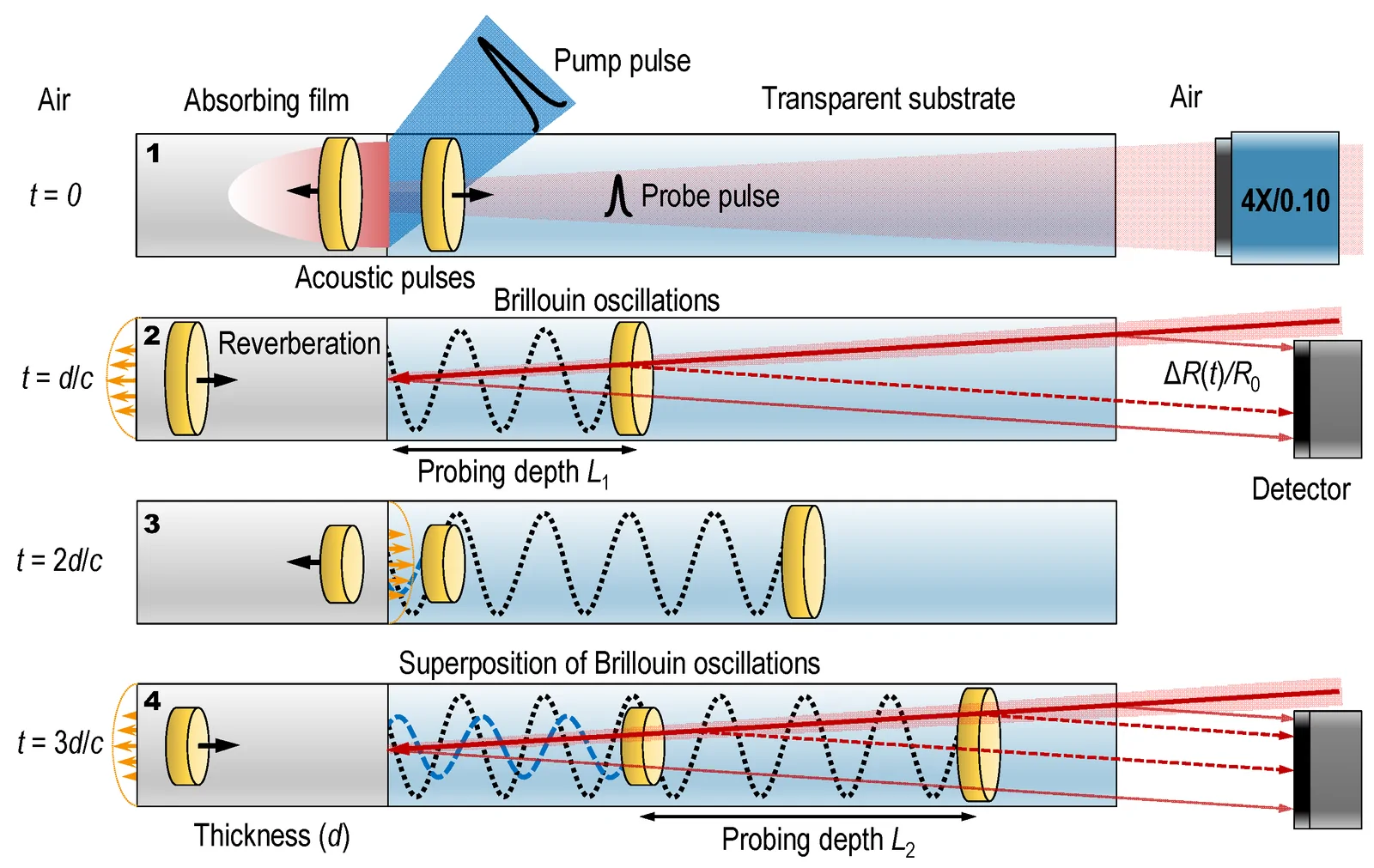
A schematic diagram for optoacoustic probing of a thin-film metal–dielectric structure by measuring transient reflectivity changes (Δ*R*(*t*)/*R*_0_) when pump and probe laser pulses are focused through the transparent substrate onto the substrate–film interface (back-pump/back-probe configuration). The probe beam is incident normally on the substrate–film interface; oblique incidence is shown solely to visualize the probe beam reflections from several stationary interfaces (air–substrate and substrate–film), and one or several moving interfaces—acoustic pulses, which simultaneously interfere on the detector. Brillouin oscillations from each acoustic pulse are shown with dotted and dashed sinusoidal lines. The resulting Δ*R*(*t*)/*R*_0_ signal represents the time-resolved superposition of individual Brillouin oscillations. Probing depths, *L*_1_ and *L*_2_, denote the thicknesses of the substrate material at the specified depths probed by a single (*L*_1_) and two (*L*_2_) acoustic pulses.

A Ti:sapphire sub-terawatt, femtosecond laser system based on chirped-pulse amplification (Legend, Coherent, USA) generating 60 fs (at FWHM) pulses at a fundamental wavelength of 793 nm and at a repetition rate of 1 kHz was used. The detection of laser-induced, propagating picosecond acoustic pulses in both materials via time-resolved measurements of transient reflectivity changes (Δ*R*(t)/*R*_0_) was carried out using an all-optical pump-probe setup with balanced, phase-sensitive detection [80]. As shown in Figure 1, the pump and probe laser pulses are focused through the substrate onto the substrate–film interface (back-pump/back-probe configuration). The 150 fs pump pulse at 396 nm (second harmonic) was focused at 45° to a 130 × 93 μm spot (at 1/e level) using a plano-convex lens with a focal length of 200 mm. The 60 fs probe at 793 nm was focused at normal incidence to a 15 μm diameter spot (at 1/e level) using a 4×/0.10 achromatic micro-objective at the center of the pump beam. The Δ*R*(t)/*R*_0_ signal was measured over a time delay range from −3 to 300 ps in the maximum possible range of non-destructive absorbed fluences (*F*_abs_) of pump pulses ranging from 1.7 to 10.3 mJ/cm^2^.

The transient reflectivity signal was measured in a balanced mode with two silicon photodiodes (DET100A/M, Thorlabs, USA) and sent to the differential input of a lock-in amplifier (SR830 DSP, Stanford Research Systems, USA). The lock-in amplifier was tuned to the 0.5 kHz repetition rate of the pump pulses modulated by a mechanical chopper (MC2000B, Thorlabs, USA) with a 60-slot disk (MC1F60, Thorlabs, USA). With a time constant of 30 ms and a filter slope of 24 dB/octave, the equivalent noise bandwidth was 2.6 Hz, yielding an effective signal averaging time of 300 ms. It should be emphasized that the maximum absorbed fluence (*F*_abs_) withstood by the sample in the experiment was *F*_abs_ = 10.3 mJ/cm^2^ at a pulse repetition rate of 0.5 kHz (the ultimate thermomechanical loads). A further increase in the fluence led to sample degradation and destruction.

To experimentally demonstrate the effect of the thickness of an optoacoustic transducer film on the resulting waveform of Brillouin oscillations within a glass substrate, another sample consisting of a 150 μm borosilicate-glass-supported submicroscale (0.92 μm) nickel film was used.

## 3. Results

**Figure 2:**
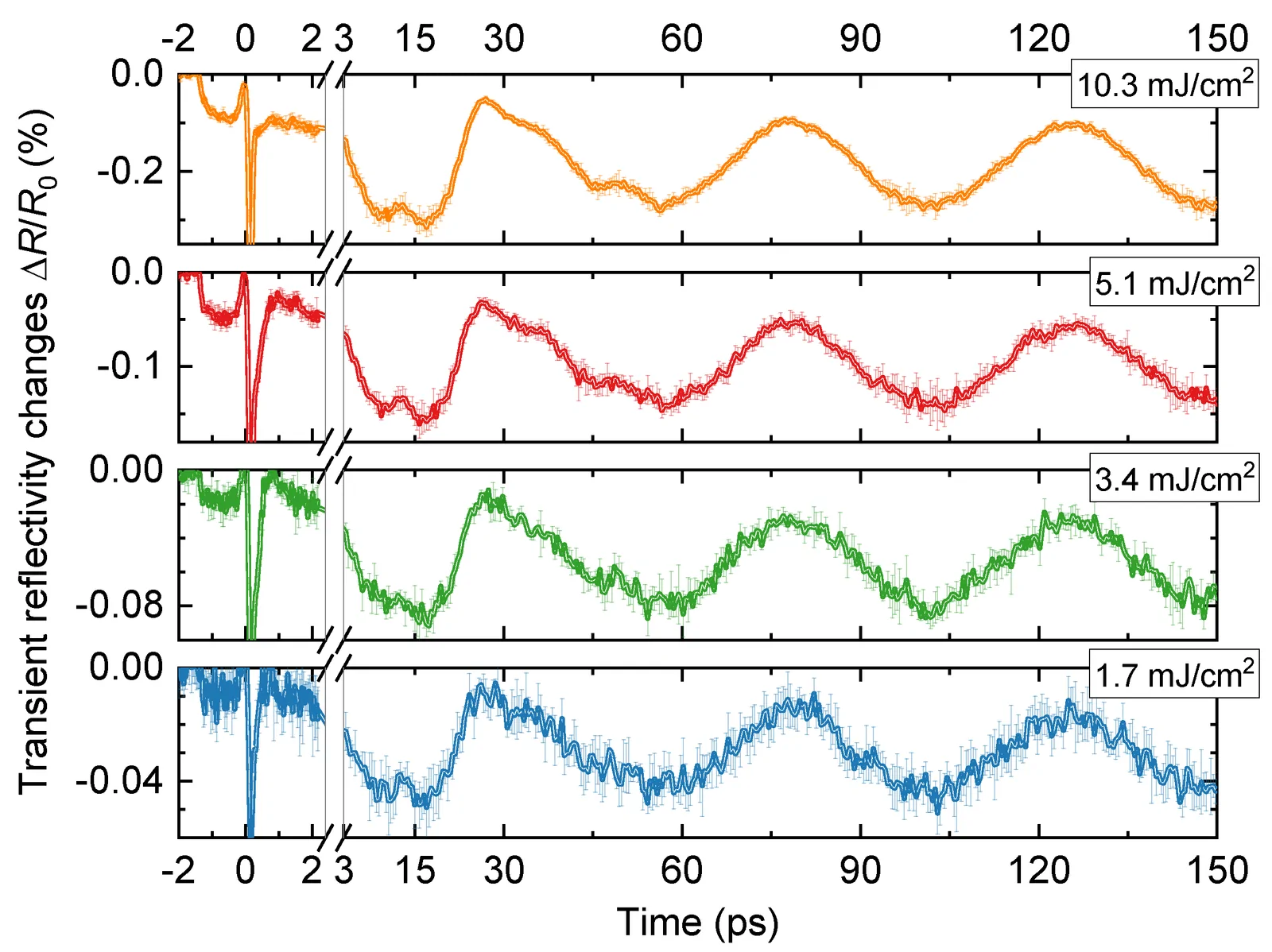
Transient reflectivity changes Δ*R*(*t*)/*R*_0_ (in %) of a borosilicate glass-supported 70 nm thick nickel film in the back-pump/back-probe configuration over the pump-probe delay range (Δ*t*_delay_) from −2 to 150 ps at absorbed pump fluences of *F*_abs_ = 1.7, 3.4, 5.1 and 10.3 mJ/cm^2^; the time step corresponds to Δ*t*_1_ = 30 fs and Δ*t*_2_ = 0.4 ps for the delay ranges from −2 to 2.2 ps and from 2.2 to 150 ps, respectively.

Figure 2 shows the original experimental data of the transient reflectivity changes Δ*R*(*t*)/*R*_0_ in the back-pump/back-probe configuration of the glass-supported 70 nm nickel film for the absorbed fluences of the pump pulse *F*_abs_ = 1.7, 3.4, 5.1, and 10.3 mJ/cm^2^ over the pump-probe delay range (Δ*t*_delay_) from −2 to 150 ps. Using an optical delay line (LTS300/M, Thorlabs, USA), the time step was set to Δ*t*_1_ = 30 fs from −2 to 2.2 ps and to Δ*t*_2_ = 0.4 ps from 2.2 to 150 ps. As a result, all the nuances of the ultrafast-laser-induced transient reflectivity changes were captured. The initial moment (*t* = 0), representing the zero-time delay, corresponds to the maximum of the cross-correlation function between the pump and probe pulses. For each pump fluence, the Δ*R*(*t*)/*R*_0_ signal was measured several times. Consequently, the averaged Δ*R*(*t*)/*R*_0_ signals with the corresponding values of the root-mean-square deviation are shown in Figure 2. Note that the transient reflectivity changes are shown in percent (%) meaning that, for example, at Δ*t*_delay_ = 15 ps for *F*_abs_ = 1.7, 3.4, 5.1 and 10.3 mJ/cm^2^ the signal reaches negative relative changes in Δ*R*/*R*_0_ = 0.045% (or 4.5 × 10^−4^), 0.084% (8.4 × 10^−4^), 0.15% (1.5 × 10^−3^) and 0.3% (3.0 × 10^−3^), respectively. Note also that the Δ*R*(*t*)/*R*_0_ signals are in the negative domain (Δ*R*/*R*_0_ < 0) at the probing wavelength of λ_probe_ = 793 nm, which indicates lattice heating of nickel [81,82]. At Δ*t*_delay_ > 1 ps the measured transient reflectivity changes normally encode the time-dependent evolution of the thermal background, which is usually described by a multi-phase exponential function that includes the heating and cooling phases. However, an ultrafast transient change in reflectivity in the negative domain (Δ*R*/*R*_0_ < 0) immediately after laser excitation (at zero-time delay, at Δ*t*_delay_ < 1 ps) at similar absorbed pump fluences is not characteristic of nickel material [80] (see Appendix A for further details).

Now, along with the thermal background which sets the transient reflectivity changes for nickel at λ_probe_ = 793 nm into the negative domain (Δ*R*/*R*_0_ < 0), the propagation of acoustic pulses both in the nickel film and the glass substrate causes periodic changes in transient reflectivity in both the positive (Δ*R*/*R*_0_ > 0) and negative (Δ*R*/*R*_0_ < 0) domains. Within the pump pulse fluence range from 1.7 to 10.3 mJ/cm^2^, high-amplitude, laser-induced bipolar acoustic pulses with a central frequency in the gigahertz range—characterized by compression and rarefaction regions—are generated in the nanoscale nickel film and the borosilicate glass substrate with peak positive and negative pressures ranging from 1.1 to 6.5 GPa and from 0.2 to 1.2 GPa, in the film and substrate, respectively [32]. Under high-rate loading (10^8^ s^−1^), the fracture of the borosilicate glass surface above a tensile stress threshold of 6 GPa is observed, which is characterized by the ejection of material and radial cracking [83]. Converging surface acoustic waves (SAWs) were generated by focusing high-energy 200 ps laser pulses onto sacrificial gold rings deposited onto the sample surface.

In Figure 2, at a delay time of Δ*t*_delay_ > 3 ps, one can clearly observe high-amplitude, sinusoidal reflectivity oscillations for all the fluences. These oscillations have a constant period of around 46.6 ps, and their amplitude increases with pump fluence. These oscillations are referred to as Brillouin oscillations and arise from the Brillouin scattering of optical probe radiation by acoustic pulses propagating within the glass substrate. However, the transient reflectivity changes due to the propagation of acoustic pulses in the nickel film are not clearly seen in the experimental data.

Now, let us look at Figure 1 to better understand the processes involved in the generation and propagation of acoustic pulses in a thin-film metal–dielectric structure [84,85]. This structure consists of a glass substrate covered by a nanoscale metal film that acts as an optoacoustic transducer. Here, the back-pump/back-probe configuration is used, meaning that the pump and probe laser pulses are focused through the transparent substrate onto the film–substrate interface.

Upon absorption of the optical energy of the pump laser pulse within the skin layer of the metal nanoscale film, the localized and rapid temperature increase causes the material to expand thermoelastically. The generated thermoelastic stress launches a longitudinal acoustic strain pulse (acoustic pulse) which, upon interaction with the film–substrate interface, partially reflects back into the film and partially transmits into the substrate (according to the acoustic impedance mismatch). As a result, two acoustic pulses travel from the irradiated film–substrate interface in opposite directions (panel 1 in Figure 1). One acoustic pulse travels through the nanoscale film toward the free air–film interface, while the other propagates into the substrate and travels toward the other free substrate–air interface. The latter counter-propagates with the incident optical probe radiation. The acoustic pulse in both materials has a bipolar profile consisting of a leading positive-pressure region (compression) followed by a negative-pressure region (tension). This is reasonable for absorbing films (acting as optoacoustic transducers) with thicknesses (*d*) exceeding the optical penetration and electronic heating depth (δ) of the ultrashort laser pulse (that is, *d* > *δ*).

The acoustic pulse propagating within the nanoscale film bounces back and forth between the film’s boundaries (air–film and film–substrate interfaces). As a result, it creates a series of acoustic echoes at these boundaries. While experiencing total reflection at the air–film interface, the acoustic pulse partially transmits into the substrate. Consequently, apart from the initially generated acoustic pulse, a train of acoustic echoes with a decreasing amplitude (determined, for example, by acoustic and diffuse mismatch theories) and a round-trip time *T_echo_* = 2*d*/*v_g_* (where *d* is the film thickness and *v_g_* is the acoustic group velocity) leaks into the substrate (panels 3 and 4 in Figure 1). This phenomenon has a significant effect on the detected Δ*R*(*t*)/*R*_0_ signal of Brillouin oscillations and has not been duly addressed in previous studies.

In materials transparent to the probing laser radiation, the propagating acoustic pulses are detected due to the interference of the probe light reflected from stationary interfaces (e.g., substrate–air and film–substrate) with the probe light scattered by a moving interface, which is the acoustic pulse itself. The probe light scattered backward by a counter-propagating acoustic pulse, which causes small localized changes in density (Δ*ρ*/*ρ*_0_) within the material, has a low amplitude, but is detected upon interfering with a higher-amplitude probe light reflected from the sample’s stationary boundaries (optical heterodyning).

It is also worth noting that as the scattering of electromagnetic waves by elastic vibrations in a material is inelastic, the probing light reflected off a counter-propagating acoustic pulse exhibits an anti-Stokes frequency shift [44,45], which means that the frequency of the reflected light increases. Brillouin scattering is characterized by a frequency shift Δ*f*_BO_ in the gigahertz range [15,86]. For the experimental data in Figure 2, as Δ*f*_BO_ = 21.5 ± 0.1 GHz (*T*_BO_ = 46.6 ± 0.2 ps), the reflected light at the central wavelength of λ = 793 nm (*f* = 378.048 THz) is frequency-upshifted by 0.045 nm to 792.955 nm (Δ*f*_BO_/*f* = 5.687 × 10^−3^%).

However, the time-domain Brillouin scattering of the optical probing radiation by the initially generated acoustic pulse occurs over a relatively short time interval, namely for Δ*t*_delay_ < 2*d*/*v_g_* (panels 1 and 2 in Figure 1). For example, for nanoscale nickel films of 25, 50, 75 and 100 nm in thickness and a speed of sound of 5.5 nm/ps, this regime is observed at *t* < 9.1, 18.2, 27.3 and 36.4 ps, respectively, which is significantly less than, or comparable to, half or the full period of Brillouin oscillations (*T*_BO_ = 46.6 ps) in glass at normal incidence of the probe light at *λ*_probe_ = 793 nm (*T*_BO_ = λ_probe_/(2*nν*cos*θ*), where *λ*_probe_ is the wavelength of the probing optical radiation, *ν* and *n* are the velocity of the longitudinal acoustic wave and the refractive index of the substrate, and *θ* is the angle between the direction of propagation of the acoustic pulse and the incident probing optical radiation). Therefore, the film’s thickness determines the depth of the substrate material probed by a single acoustic pulse (denoted as probing depth *L*_1_ in Figure 1). Consequently, in the time range 2*d*/*v_g_* < *t* < 4*d*/*v_g_* (beginning with *t* ≥ 9.1, 18.2, 27.3 and 36.4 ps), time-domain Brillouin scattering occurs simultaneously on two acoustic pulses (panels 3 and 4 in Figure 1), and so on. As a result, at times *t* > 2*d*/*v_g_*, the detected signal of Brillouin scattering in the substrate is a superposition of individual signals of Brillouin scattering from every acoustic pulse (panels 3 and 4 in Figure 1). Thus, in the time range 2*d*/*v_g_* < *t* < 4*d*/*v_g_*, the deeper layers of the substrate material are probed by two acoustic pulses (denoted as probing depth *L*_2_ in Figure 1).

As the initially generated acoustic pulse (panels 1 and 2 in Figure 1) has the highest-pressure amplitude, it makes a dominant contribution to the detected Brillouin scattering signal (i.e., the highest amplitude of BO). Thus, the amplitude of BO caused by each subsequent acoustic echo leaking into the substrate decreases proportionally. Governed mainly by the acoustic impedance mismatch between the two contacting materials, the smaller the mismatch between impedances, the larger the amplitude of successive BO, and the stronger the effect of their superposition. However, under identical deposition conditions for similar metal films of varying thicknesses, this factor of acoustic impedance mismatch should remain *constant*. In contrast, the *dynamic* factor is the phase shift between individual BO, which is governed by the round-trip time of the acoustic echo (*T_echo_* = 2*d*/*v_g_*) in the nanoscale film and also depends on the probing light wavelength (as *T*_BO_ ~ λ_probe_). Thus, the resulting Brillouin scattering signal undergoes amplitude-phase modulation, which does not characterize the depth profile of the substrate material under study [55], but does represent an inherent instrumental characteristic which cannot be disregarded.

As mentioned earlier, the acoustic pulses reverberating within the nanoscale film should also contribute to the detected transient reflectivity changes upon reaching the air–film and film–substrate interfaces. However, visualizing this contribution at the optically probed film–substrate interface and reconstructing the time-resolved optical response of acoustic echoes within the film against the background of the high-amplitude sine waveform from Brillouin light scattering in the substrate requires mathematical post-processing procedures.

## 4. Discussion

To address the two above-stated problems—which are related to (1) the reconstruction of the decaying signal of transient reflectivity changes caused by acoustic pulses reverberating in the film and buried in the high-amplitude Brillouin scattering signal of the substrate, and (2) the effect of these acoustic echoes leaking into the substrate on the amplitude-phase modulation of the Brillouin scattering signal—the following approaches are proposed.

(1) The approach for reconstructing the Δ*R*(*t*)/*R*_0_ signal of reverberating acoustic pulses in the film against the background of the high-amplitude Brillouin oscillations in the substrate is based on filtering out the latter. Time-domain Brillouin oscillations generally follow a damped, constant-frequency sinusoidal waveform in a homogeneous medium. As mentioned before, the sinusoidal signal arises from optical heterodyne interference between probe light reflected from the stationary sample interfaces (such as air–glass and glass–nickel) and probe light scattered backward (in our case) by propagating coherent acoustic phonons. The oscillation frequency remains steady at the specific Brillouin frequency (*f*_BO_ = 1/*T*_BO_) defined by the sound velocity and effective probe wavelength (λ_probe_/*n*), while damping over time is due to acoustic attenuation and phonon lifetime limits in the material, as well as dephasing effects of the broadband probing radiation [62,63].

However, the detected Brillouin oscillations undergo amplitude-phase modulation in certain cases due to the superposition of individual Brillouin scattering signals from each acoustic echo pulse (panels 3 and 4 in Figure 1). This implies that one cannot simply fit the experimentally measured signal of Brillouin oscillations (which has presumably undergone amplitude-phase modulation) with a damped monochromatic sine waveform if the fitting time range covers the range where the acoustic echoes have already leaked into the substrate (compare Figure A2 and Figure A3 shown Appendix A). In our case, for the 70 nm nickel film, the first echo pulse leaks into the substrate in the time range from *T*_echo_ = 23.3 to 28 ps (as calculated for sound velocities ranging from 5 to 6 nm/ps). Since the calculated *T*_echo_ is close to half the period of the Brillouin oscillations (*T*_BO_ = 46.6 ps), the fitting would not be very accurate over such a short time interval. Fitting over a wider time range would inherently cover the range of the amplitude-phase modulated Brillouin scattering signal, which could result in a distorted reconstruction of the sought-for echo-induced transient reflectivity changes and residual Brillouin oscillations.

Here, filtering out the Brillouin oscillations from the experimental Δ*R*(*t*)/*R*_0_ signal (Figure 2) is achieved using a zero-phase, high-pass, symmetric three-tap finite impulse response (FIR) filter defined as follows:

*g*(*t*) = 0.5*f*(*t*) − 0.25*f*(*t* − *T*/2) − 0.25*f*(*t* + *T*/2), (1)

where *f*(*t*) is the input signal, *g*(*t*) is the filtered output, and *T* serves as the characteristic time period defining the tap delay interval Δ*t* = *T*/2. The filter coefficients can be expressed as a vector *m* = [−0.25, 0.5, 0.25] for the backward-looking (*t − T*/2), central (*t*), and forward-looking (*t* + *T*/2) taps. Because *m*(*τ*) *= m*(−*τ*), the filter is strictly symmetric around the central tap (*t*). The forward-looking tap *f*(*t* + *T*/2) introduces non-causality, meaning that this formulation is ideally suited for offline batch processing where the entire data sequence is accessible. The key advantages of this filter are its zero-phase shift (linear phase), complete direct current (DC) rejection, unconditional stability, and computational simplicity.

In signal processing and numerical analysis, this filter is a discrete approximation of a negative second-order differential operator designed to isolate (or enhance) high-frequency and block low-frequency (DC) components. The expression can be rewritten by grouping terms into a standard central finite difference format:

(2) $$g(t)=-\frac{1}{4}\left[f\left(t+\frac{T}{2}\right)-2f\left(t\right)+f\left(t-\frac{T}{2}\right)\right]$$

The second-order central difference approximation for the second derivative with a truncation error of *O*(*h*^2^) (substituting a time step *h* = *T*/2) is given by the formula:

(3) $$\frac{\partial^{2}f}{\partial t^{2}}\approx \frac{f\left(t+h\right)-2f\left(t\right)+f\left(t-h\right)}{h^{2}}$$

Substituting a time step *h* = *T*/2 into Expression (3), one can obtain the Expression (1) for *g*(*t*) behaves like a scaled, inverted second-order derivative operator:

(4) $$g(t)\approx -\frac{T^{2}}{16}\frac{d^{2}f(t)}{dt^{2}}$$

Consequently, this operator also acts as a high-pass curvature detector, tuned to a specific period *T* (or frequency *f =* 1/*T*), which attenuates slowly varying changes while amplifying rapid fluctuations within the input signal *f*(*t*).

It can also be noted that for an input function *f*(*t*) containing (or consisting of) a pure sinusoidal waveform with a period *T* (i.e., *f*(*t*) = *A*sin[(2π/*T*)*t* + *ϕ*], where *A* is the amplitude, *T* = 1/*f* = 2π/ω is the period, and *ϕ* is the phase in radians), the filtered output *g*(t) in Expression (1) results in the reconstructing of the function *f*(*t*) up to the original amplitude *A*:

(5) $$g(t)=A\sin\left[\left(\frac{2\pi }{T}\right)t+\varphi \right]$$

To convert a time-domain signal *g*(*t*) into its frequency-domain spectrum *F*(*ω*), one can apply the continuous-time Fourier transform:

(6) $$F\left\{f(t)\right\}=F(\omega )=\int_{-\infty }^{\infty }f(t)\exp\left(-\mathrm{i\omega t}\right)\mathrm{dt}$$

The Fourier transform is a linear operation, and it follows the time-shifting property:

ℱ{*f*(*t* ± *a*)} = *F*(*ω*)exp(±*iωa*) (7)

Applying Expressions (6) and (7) to Expression (1), one can obtain the total spectrum *G*(*ω*) for the function *g*(*t*):

(8) $$G(\omega )=F(\omega )\left[0.5-0.25\exp\left(-\mathrm{i\omega}\frac{T}{2}\right)-0.25\exp\left(\mathrm{i\omega}\frac{T}{2}\right)\right]$$

Using Euler’s identity exp(*ix*) + exp(−*ix*) = 2cos(*x*), the final expression for the total spectrum *G*(*ω*) is given as follows:

(9) $$G(\omega )=F(\omega )\left[0.5\left(1-\cos\left(\frac{\mathrm{\omega T}}{2}\right)\right)\right]$$

Therefore, the frequency response *H*(*ω*) = *G*(*ω*)/*F*(*ω*) using the half-angle trigonometric identity 0.5(1 − cos(*x*)) = sin^2^(*x*/2) is derived as follows:

(10) $$H(\omega )=\sin^{2}\left(\frac{\mathrm{\omega T}}{4}\right)$$

As the frequency response *H*(*ω*) is purely real and non-negative (*H*(*ω*) ≥ 0) for all frequencies *ω = 2π/T*, the filter introduces a zero-phase shift, which guarantees the perfect temporal alignment between in the input *f*(*t*) and output *g*(*t*) functions without any group delay distortion. Then, substituting the harmonic frequencies *ω = kω*_0_ *=* 2*πk/T* (where *ω*_0_ *=* 2*π/T* is the fundamental frequency and *k* is a positive integer) into Expression (10) for the frequency response *H*(*ω*) yields *H*(*ω*) = sin^2^(*kπ*/2). For even harmonics (*k* = 0, 2, 4, …) *H*(*ω*) = 0, which means that the filter completely suppresses the DC component, low-frequency baseline trends, and all even harmonics. The maximum gain *H*(*ω*) = 1 occurs for odd harmonics (*k* = 1, 3, 5, …), including the desired fundamental frequency *ω*_0_ *=* 2*π/T* with a zero-phase shift.

**Figure 3:**
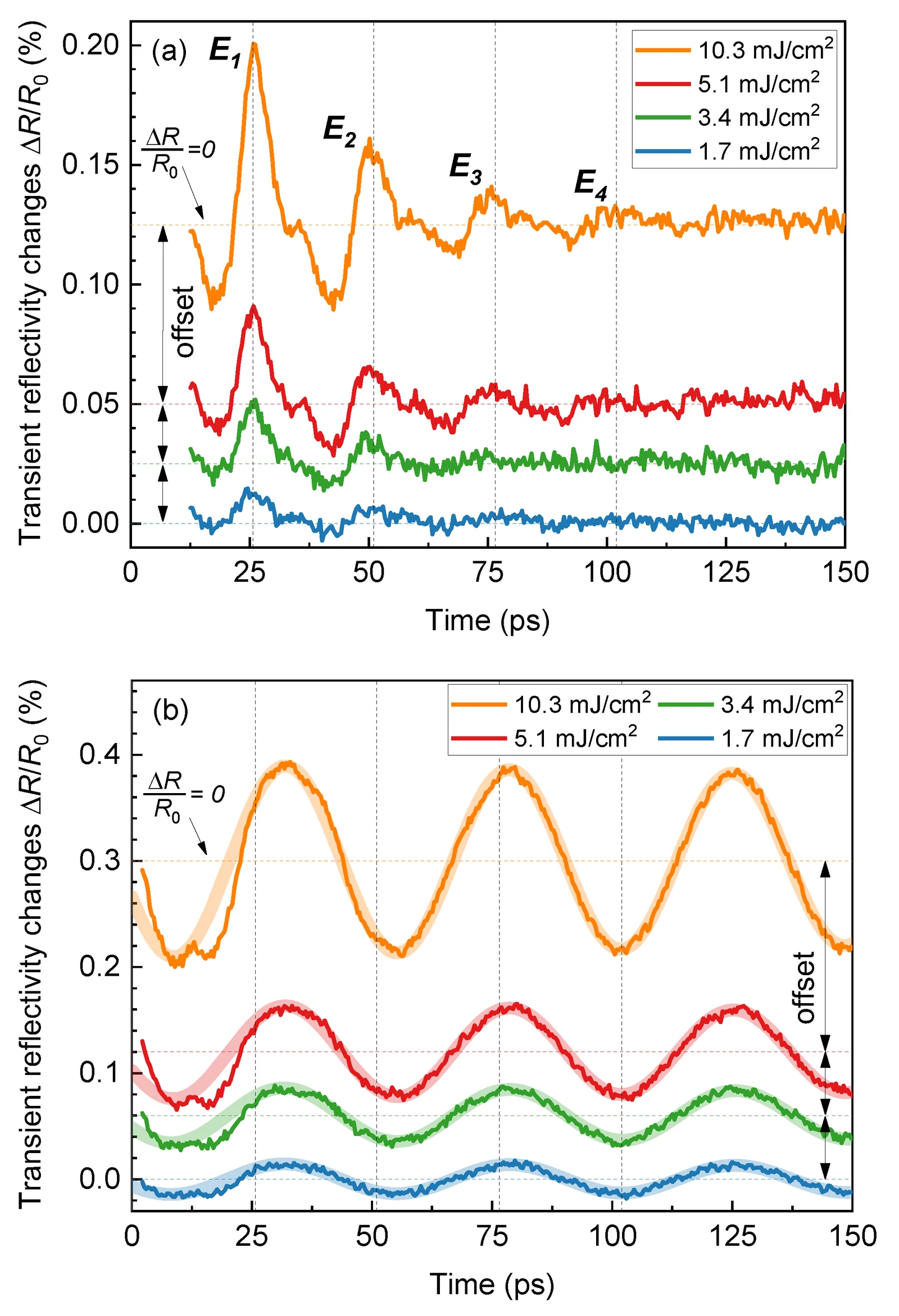
(**a**) Reconstructed transient reflectivity changes Δ*R*(*t*)/*R*_0_ caused by reverberating acoustic pulses in the film (an acoustic echo pulse) at a round-trip time of *T*_echo_ = 25.6 ps and detected at the substrate–film interface against a high-amplitude background of Brillouin oscillations in the substrate; (**b**) reconstructed transient reflectivity changes Δ*R*(*t*)/*R*_0_ caused by propagating acoustic pulses in the substrate and detected as Brillouin oscillations with a period of *T*_BO_ = 46.6 ps. The experimental data are shown at absorbed pump fluences of *F*_abs_ = 1.7, 3.4, 5.1, and 10.3 mJ/cm^2^. The symbols *E*_1_, *E*_2_, *E*_3_, and *E*_4_ (along with vertical dashed lines) stand for the acoustic echo pulses. The offset of each curve corresponds proportionally to the increasing *F*_abs_. The levels of Δ*R*(*t*)/*R*_0_ = 0 are shown with horizontal dashed lines.

In practice, to filter out the Brillouin oscillations from the original experimental Δ*R*(*t*)/*R*_0_ signal (Figure 2) using the FIR filter (Expression (1)), one should first specify the period of the Brillouin oscillations (*T*). The output function *g*(*t*) then calculates a linear combination of the functions *f*(*t* − *T*/2), *f*(*t*) and *f*(*t* + *T*/2), which are the original experimental Δ*R*(*t*)/*R*_0_ signals at time points *t* − *T*/2, *t* and *t* + *T*/2, respectively. The resulting output signals *g*(*t*), obtained by applying the filter to the original experimental signals Δ*R*(*t*)/*R*_0_ at *F*_abs_ = 1.7, 3,4, 5.1 and 10.3 mJ/cm^2^ for the predetermined period of Brillouin oscillations *T* = *T*_BO_ = 46.6 ps (using the Fast Fourier Transform in OriginPro, Version 2021), are shown in Figure 3b. These reconstructed sinusoidal transient reflectivity changes in the time interval up to 150 ps are referred to as Brillouin oscillations and generally caused by propagating acoustic pulses in the substrate. Subsequently, subtracting these Brillouin oscillations signals (which are the *g*(*t*) signals at *T*_BO_ = 46.6 ps) from the original experimental Δ*R*(*t*)/*R*_0_ signals results in the decaying non-sinusoidal periodic signals shown in Figure 3a. These reconstructed non-sinusoidal transient reflectivity changes are generally caused by reverberating acoustic pulses in the film with a round-trip time of *T*_echo_ = 25.6 ps and are detected at the substrate–film interface. Up to four acoustic echoes at 25.6, 51, 76.5 and 102 ps marked as *E*_1_, *E*_2_, *E*_3_, and *E*_4_ in Figure 3a, are observed.

At the highest fluence *F*_abs_ = 10.3 mJ/cm^2^, the maximum Δ*R*(*t*)/*R*_0_ signal corresponding to the first echo at 25.6 ps reaches 0.08%, while the last discernible fourth echo at 102 ps reaches only 0.004% which is close to the standard deviation of 0.0028% from the mean value of the original Δ*R*(*t*)/*R*_0_ signal at negative pump-probe delays after the averaging of several datasets. At the lowest fluence *F*_abs_ = 1.7 mJ/cm^2^, the maximum Δ*R*(*t*)/*R*_0_ signal corresponding to the first echo at 25.6 ps reaches only 0.012%, while the last discernible third echo at 76.5 ps reaches 0.0026%, which corresponds to the standard deviation of 0.0026% for the current averaged dataset and thus eventually prevents observation of subsequent echoes at such low laser excitation.

To further improve the measurement accuracy, one has to increase the number of datasets *N* (as the standard error decreases proportionally to $1/\sqrt{N}$). Thus, for example, to cut the error in half, the number of datasets *N* must be increased fourfold. However, to cut the error by an order of magnitude, *N* must be increased by two orders of magnitude, which is not practical at the relatively low repetition rate (0.5 kHz) of the pump pulses. For the experimental data shown in Figure 2, the number of averaged datasets was *N* = 3, which allowed for the experimental observation of up to three echoes at *F*_abs_ = 1.7 and 3.4 mJ/cm^2^, and up to four echoes at *F*_abs_ = 5.1 and 10.3 mJ/cm^2^. To record a single dataset in the time interval from −2 to 150 ps took around 3 min; thus, recording *N* = 3 datasets took around 9 min. Increasing the number of datasets fourfold (*N* = 3 × 4) and by two orders of magnitude (*N* = 3 × 10^2^) would result in 0.6 and 15 h of total recording time, respectively.

Now, based on the reconstructed Δ*R*(*t*)/*R*_0_ signals, induced by reverberating acoustic pulses (Figure 3a), the longitudinal sound velocity (*c*_L_) in the nanoscale nickel film, which gives the measured *T*_echo_ = 25.6 ps, is estimated as *c*_L_ = 2*d*/*T*_echo_ = 5.47 ± 0.02 nm/ps. This value agrees with the calculated values of longitudinal sound velocity for nano- and microcrystalline nickel films 5.35–5.73 nm/ps ($c_{L}=\sqrt{3B\left[\left(1-\nu \right)/\left(1+\nu \right)\right]/\rho }$, where ν = 0.31 is the Poisson ratio, ρ = 8900 kg/m^3^ is the density, and *B* = 161–185 GPa is the range of the bulk modulus [87]). It is also within the experimentally measured range of longitudinal sound velocities from 5.5 to 6 nm/ps for nickel films of various thicknesses (from 120 to 300 nm) obtained using picosecond acoustics [16,18,88,89,90]. However, for certain nickel films with thicknesses of 10 and 200 nm, the experimental values were found to drop to 3.8–4.5 nm/ps [91,92]. While fundamentally defined by the ratio of the longitudinal elastic modulus to the mass density, the effective acoustic velocity in deposited (sputtered) metallic films exhibits pronounced dependencies on processing-controlled parameters. Variations in substrate temperature, ambient gas pressure, and deposition rate directly alter grain size and orientation, residual stress, porosity (defects), composition, and impurities. As a result, deposited films of the same thickness demonstrate different electrical, optical, mechanical, acoustic, magnetic, and thermal behaviors. Therefore, the non-destructive evaluation of the longitudinal sound velocity is of considerable importance for each individual deposited film, particularly those of nanoscale thickness.

**Figure 4:**
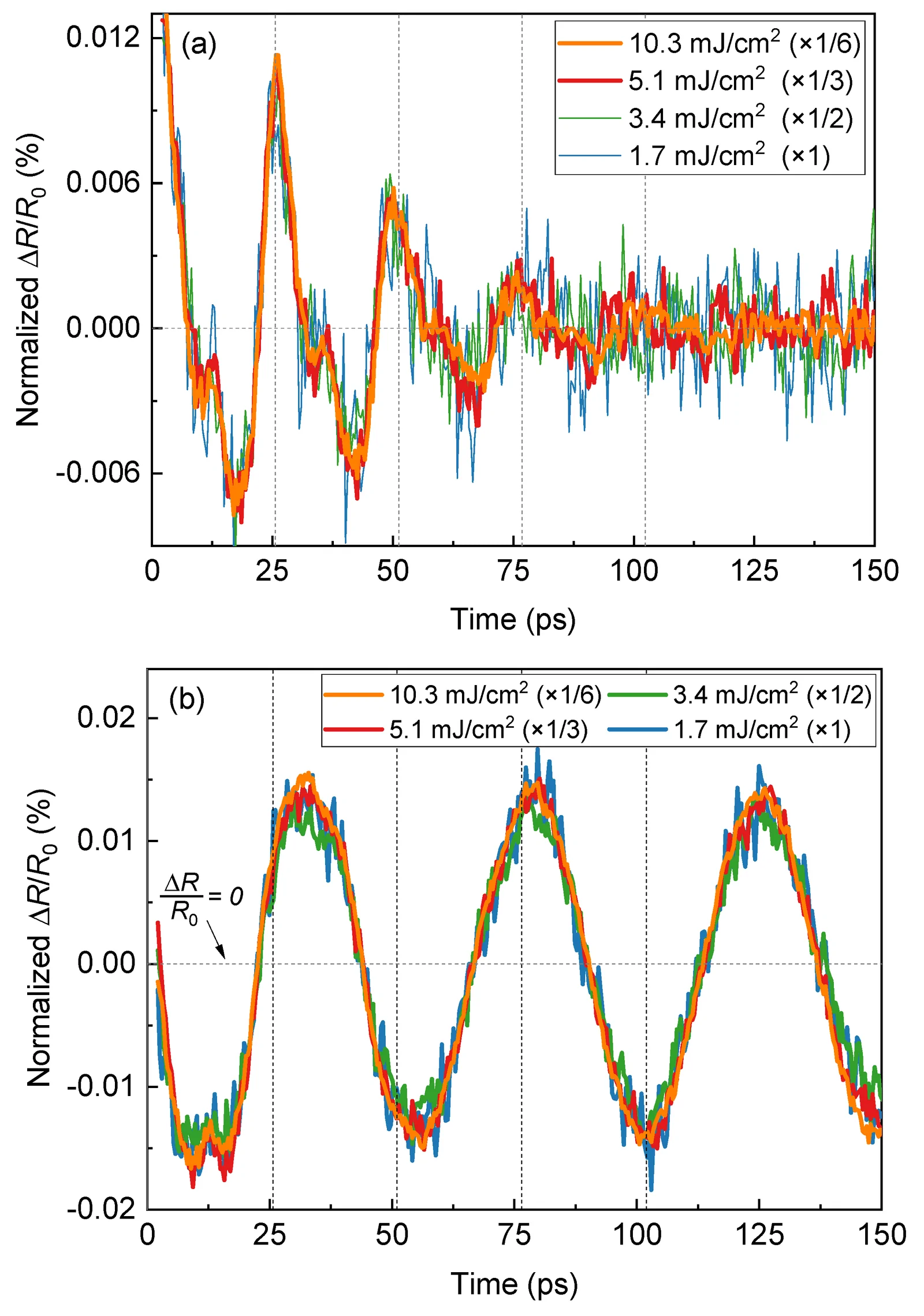
Normalized reconstructed transient reflectivity changes Δ*R*(*t*)/*R*_0_ caused by (**a**) reverberating acoustic pulses in the film and (**b**) propagating acoustic pulses in the substrate. The experimental curves are normalized to match the increasing absorbed laser fluences: *F*_1_ = 1.7, *F*_2_ = 3.4, *F*_3_ = 5.1, and *F*_4_ = 10.3 mJ/cm^2^, and are multiplied by factors of *F*_1_/*F*_1_ = 1, *F*_1_/*F*_2_ = 1/2, *F*_1_/*F*_3_ = 1/3, and *F*_1_/*F*_4_ = 1/6. The vertical dashed lines correspond to the acoustic echoes at the film–substrate interface.

To compare the normalized Δ*R*(*t*)/*R*_0_ signals at different absorbed laser fluences *F*_abs_ (ranging from 1.7 to 10.3 mJ/cm^2^), the contributions from reverberating acoustic pulses in the film and propagating acoustic pulses in the substrate are shown in Figure 4a and Figure 4b, respectively.

The experimental curves are normalized in accordance with the increasing absorbed fluences: *F*_1_ = 1.7, *F*_2_ = 3.4, *F*_3_ = 5.1, and *F*_4_ = 10.3 mJ/cm^2^, and multiplied by the corresponding factors *F*_1_/*F*_1_ = 1, *F*_1_/*F*_2_ = 1/2, *F*_1_/*F*_3_ = 1/3, and *F*_1_/*F*_4_ = 1/6. As seen from Figure 4a, the waveforms of the Δ*R*(*t*)/*R*_0_ signals caused by reverberating acoustic pulses in the film demonstrate no significant changes with increasing laser fluence. Specifically, no waveform variations or relative time delays of the echo peaks [93] are observed. This is an important experimental observation showing the conservation of the transient reflectivity signal waveform caused by reverberating acoustic pulses in the nanoscale film and detected at the substrate–film interface within this range of high laser fluences [94]. Furthermore, given the linear scaling of the Δ*R*(*t*)/*R*_0_ signals with increasing fluence, it appears that the peak values of the echoes also scale linearly with the deposited energy. This could imply that the underlying generation mechanism is in the linear thermoelastic regime, where the local temperature rise and resulting stress are directly proportional to the absorbed laser fluence. This is also supported by the linear scaling of the amplitude of Brillouin oscillations with increasing deposited energy, as shown in Figure 4b. Here, the waveforms of the Δ*R*(*t*)/*R*_0_ signals caused by propagating acoustic pulses in the substrate exhibit similar profiles and the same phase delay.

**Figure 5:**
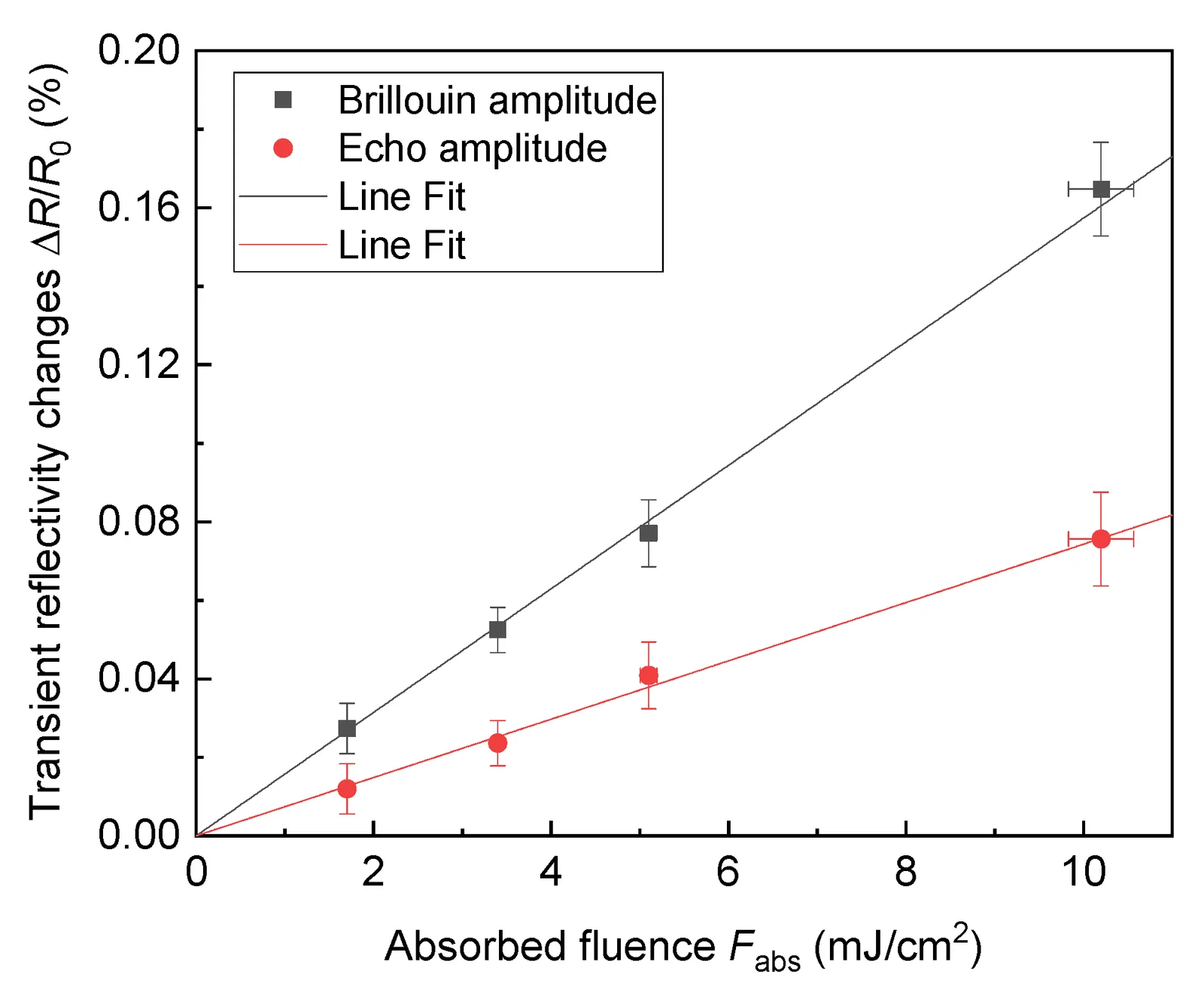
Transient reflectivity changes Δ*R*(*t*)/*R*_0_, which denote the amplitudes of Brillouin oscillations (black squares) and the first acoustic echo (red circles), are shown as a function of the absorbed laser fluence *F*_abs_. Linear fits are represented by solid lines.

Figure 5 shows the transient reflectivity changes Δ*R*(*t*)/*R*_0_, which represent the amplitude of the Brillouin oscillations *f*(*A*_BO_) and the first acoustic echo *f*(*A*_echo_) as a function of the absorbed laser fluence *F*_abs_. Both dependencies, *f*(*A*_BO_, *F*_abs_) and *f*(*A*_echo_, *F*_abs_), exhibit linear scaling from the origin but with different slopes because the optical response varies for acoustic pulses detected in the metal film versus the glass substrate. Thus, within the range of relatively high *F*_abs_ (from 1.7 to 10.3 mJ/cm^2^), considering the linear scaling of optical responses for acoustic pulses propagating in the metal film and the transparent substrate, the peak pressures in the laser-induced compression and tension regions of propagating acoustic pulses are estimated to be between 1.1 and 6.5 GPa in the nanoscale nickel film and between 0.2 and 1.2 GPa in the borosilicate glass substrate [32].

**Figure 6:**
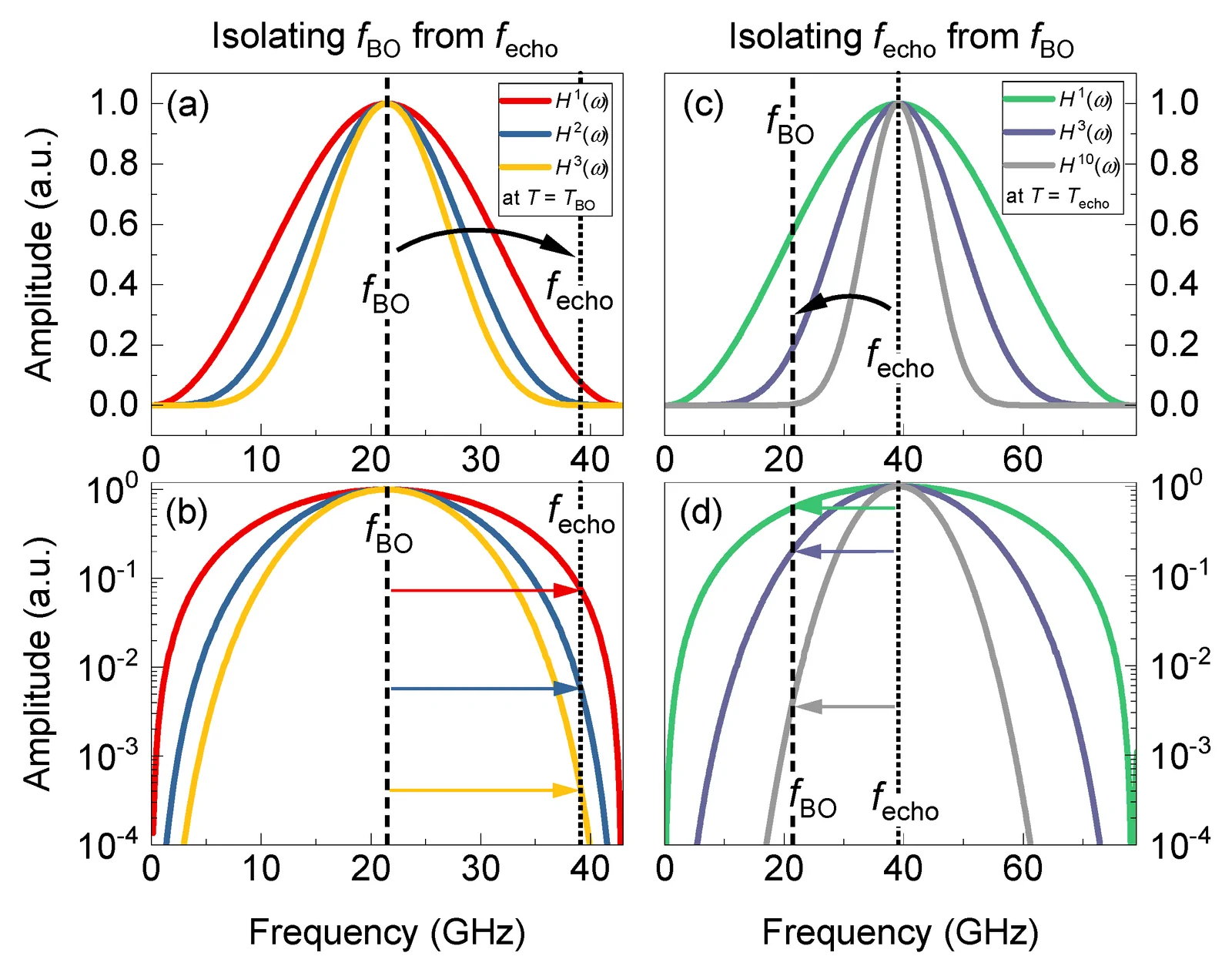
Frequency responses *H*(*ω*) of time-domain functions *g*(*t*) showing various roll-offs at the specific central frequencies *f*_BO_ = 21.5 GHz and *f*_echo_ = 39.1 GHz, which denote propagating acoustic pulses in the substrate and film, respectively. (**a**,**b**) The frequency response *H*(*ω*) at *T* = *T*_BO_ for isolating the signal of Brillouin oscillations at *f*_BO_ in the substrate from the signal of acoustic echo pulses at *f*_echo_ in the film in linear and logarithmic *Y*-scales; using *H*^1^(*ω*), *H*^2^(*ω*) and *H*^3^(*ω*) at *T* = *T*_BO_ results in a signal reduction at *f*_echo_ to levels of 0.0741, 0.0055 and 0.0004, respectively; (**c**,**d**) The frequency response *H*(*ω*) at *T* = *T*_echo_ for isolating the signal of acoustic echo pulses at *f*_echo_ in the film from the signal of Brillouin oscillations at *f*_BO_ in the substrate in linear and logarithmic Y-scales; using *H*^1^(*ω*), *H*^3^(*ω*) and *H*^10^(*ω*) at *T* = *T*_echo_ results in a signal reduction at *f*_BO_ to levels of 0.5766, 0.1917 and 0.0041, respectively.

Now, the reconstructed Δ*R*(*t*)/*R*_0_ signals, caused by propagating acoustic pulses in the substrate (Brillouin oscillations at *T*_BO_ = 46.6 ps or *f*_BO_ = 21.5 GHz) and shown in Figure 3b, can be approximated by an exponentially decaying sinusoidal function, given by:

(11) $$y\left(t\right)=A\exp\left(-t/t_{0}\right)\sin\left(\frac{2\pi }{T}\left(t-\varphi \right)\right),$$

where *φ* = 20.40 ± 0.04 ps is the phase shift, *T =* 46.57 ± 0.01 ps is the period of Brillouin oscillations, *t*_0_ = 1.7 ± 0.1 ns is the damping time constant, and *A* is the amplitude which scales depending on the absorbed fluence. It is worth noting that due to the superposition of individual Brillouin oscillations (see Figure 1), the fitting would be sensitive to the chosen fitting range. Here, a fitting range from 56 to 300 ps, which results in the minimal residual oscillations, was chosen (see Appendix A, Figure A2). As follows from Figure 3b, the maximum discrepancy between the reconstructed Δ*R*(*t*)/*R*_0_ signals and the corresponding damped sine waves is observed in the time interval Δ*t*_delay_ < 25 ps. To clarify this phenomenon, one should refer to the frequency response *H*(*ω*), which is described by Expression (10) for the FIR filter in the time domain by Expression (1) (see Figure 6).

As shown in Figure 6a, the frequency response *H*(*ω*) of Expression (10) is centered at 21.5 GHz, has a bandwidth of Δ*f* = 1/*T =* 21.5 GHz at the FWHM level, and passes around 7.4% at the frequency *f*_echo_ = 39.1 GHz (*T*_echo_ = 25.6 ps) of the reverberating acoustic pulses in the nanoscale nickel film (Figure 6b). Thus, to fully isolate the Brillouin oscillations at *f*_BO_ = 21.5 GHz in the substrate from the reverberating acoustic pulses at *f*_echo_ = 39.1 GHz in the film, one has to make a filter based on Expression (10), *H*(*ω*) = sin^2^(*ωT*/4), narrower. To make this filter narrower, while keeping the same central period *T*, one can raise the squared sine term to a higher even power, for example, *H^n^*(*ω*) = sin^2n^(*ωT*/4) where *n* > 1. This will decrease the filter passband and widen the notch/stopband region while preserving the fundamental period *T*. Figure 6a,b show the frequency responses for *n* = 2 [*H*^2^(*ω*) = sin^4^(*ωT*/4)] and *n* = 3 [*H*^3^(*ω*) = sin^6^(*ωT*/4), in addition to *n* = 1 [*H*^1^(*ω*) = sin^2^(*ωT*/4)]. As seen in Figure 6a,b, using *H*^1^(*ω*), *H*^2^(*ω*) and *H*^3^(*ω*) results in a signal reduction at *f*_echo_ = 39.1 GHz to levels of 0.0741 (7.41%), 0.0055 (0.55%) and 0.0004 (0.04%), respectively.

Now, the corresponding time-domain signals *g*^1^(*t*), *g*^2^(*t*) and *g*^3^(*t*), which match the given frequency responses *H*^1^(*ω*), *H*^2^(*ω*) and *H*^3^(*ω*), can be obtained using the inverse Fourier transform. In fact, the inverse Fourier transforms of *H^n^*(*ω*) = sin^2n^(*ωT*/4) for *n* = 1, 2, 3 consist entirely of discrete, time-shifted Dirac delta functions weighted by symmetric binomial coefficients with alternating signs, such as *g*^2^(*t*) and *g*^3^(*t*) shown below for *n* = 2 and 3:

(12) $$g^{2}(t)=\frac{3}{8}\delta \left(t\right)+\frac{1}{16}\delta \left(t-1\right)+\frac{1}{16}\delta \left(t+1\right)-\frac{1}{4}\delta \left(t-\frac{1}{2}\right)-\frac{1}{4}\delta \left(t+\frac{1}{2}\right)$$

(13) $$g^{3}(t)=\frac{5}{16}\delta \left(t\right)+\frac{3}{32}\delta \left(t-1\right)+\frac{3}{32}\delta \left(t+1\right)-\frac{15}{64}\delta \left(t-\frac{1}{2}\right)-\frac{15}{64}\delta \left(t+\frac{1}{2}\right)-\frac{1}{64}\delta \left(t-\frac{3}{2}\right)-\frac{1}{64}\delta \left(t+\frac{3}{2}\right)$$

**Figure 7:**
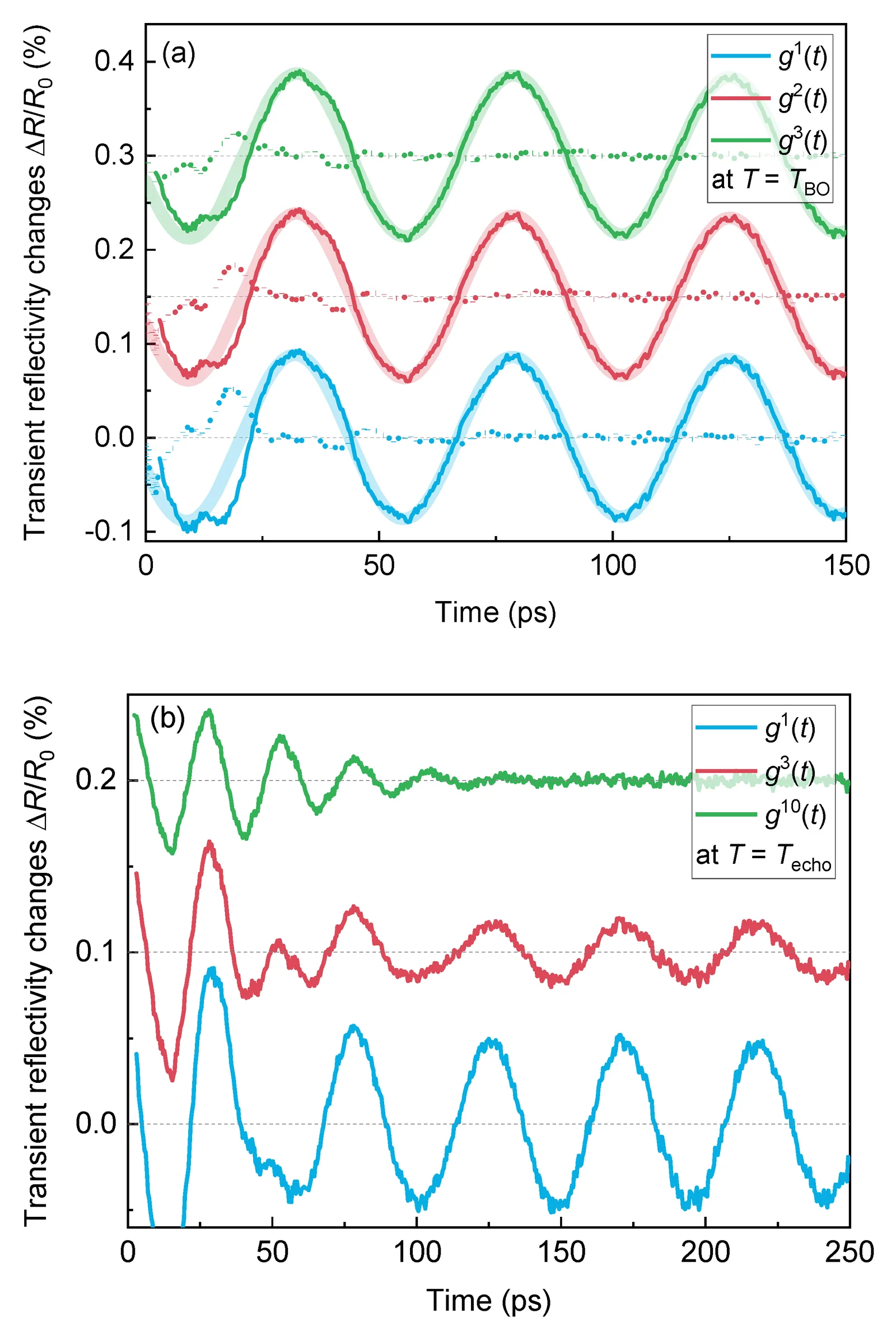
(**a**) Isolation of the Δ*R*(*t*)/*R*_0_ signal of Brillouin oscillations in the substrate (*f*_BO_ = 21.5 GHz) from the Δ*R*(*t*)/*R*_0_ signal of reverberating acoustic pulses in the film (*f*_echo_ = 39.1 GHz) for the experimental Δ*R*(*t*)/*R*_0_ signal at *F*_abs_ = 10.3 mJ/cm^2^ (Figure 2) using time-domain functions *g*^1^(*t*), *g*^2^(*t*) and *g*^3^(*t*) at *T* = *T*_BO_, whose frequency responses are shown in Figure 6a,b. The exponentially damped sinusoidal approximations are shown with thick semitransparent solid lines, and the difference between the reconstructed Δ*R*(*t*)/*R*_0_ signals and the corresponding damped sine waves are shown with short-dotted lines. (**b**) Conversely, isolation of Δ*R*(*t*)/*R*_0_ signal of reverberating acoustic pulses in the film (*f*_echo_) from the Δ*R*(*t*)/*R*_0_ signal of Brillouin oscillations in the substrate (*f*_BO_) for the experimental Δ*R*(*t*)/*R*_0_ signal at *F*_abs_ = 10.3 mJ/cm^2^ using time-domain functions *g*^1^(*t*), *g*^3^(*t*) and *g*^10^(*t*) at *T* = *T*_echo_, whose frequency responses are shown in Figure 6c,d.

Figure 7a shows the resulting output signals *g*^1^(*t*), *g*^2^(*t*) and *g*^3^(*t*) applied to the original experimental signal Δ*R*(*t*)/*R*_0_ at *F*_abs_ = 10.3 mJ/cm^2^ and at *T*_BO_ = 46.6 ps. These signals are fitted by a damped sine wave (Expression (11)).

**Figure 8:**
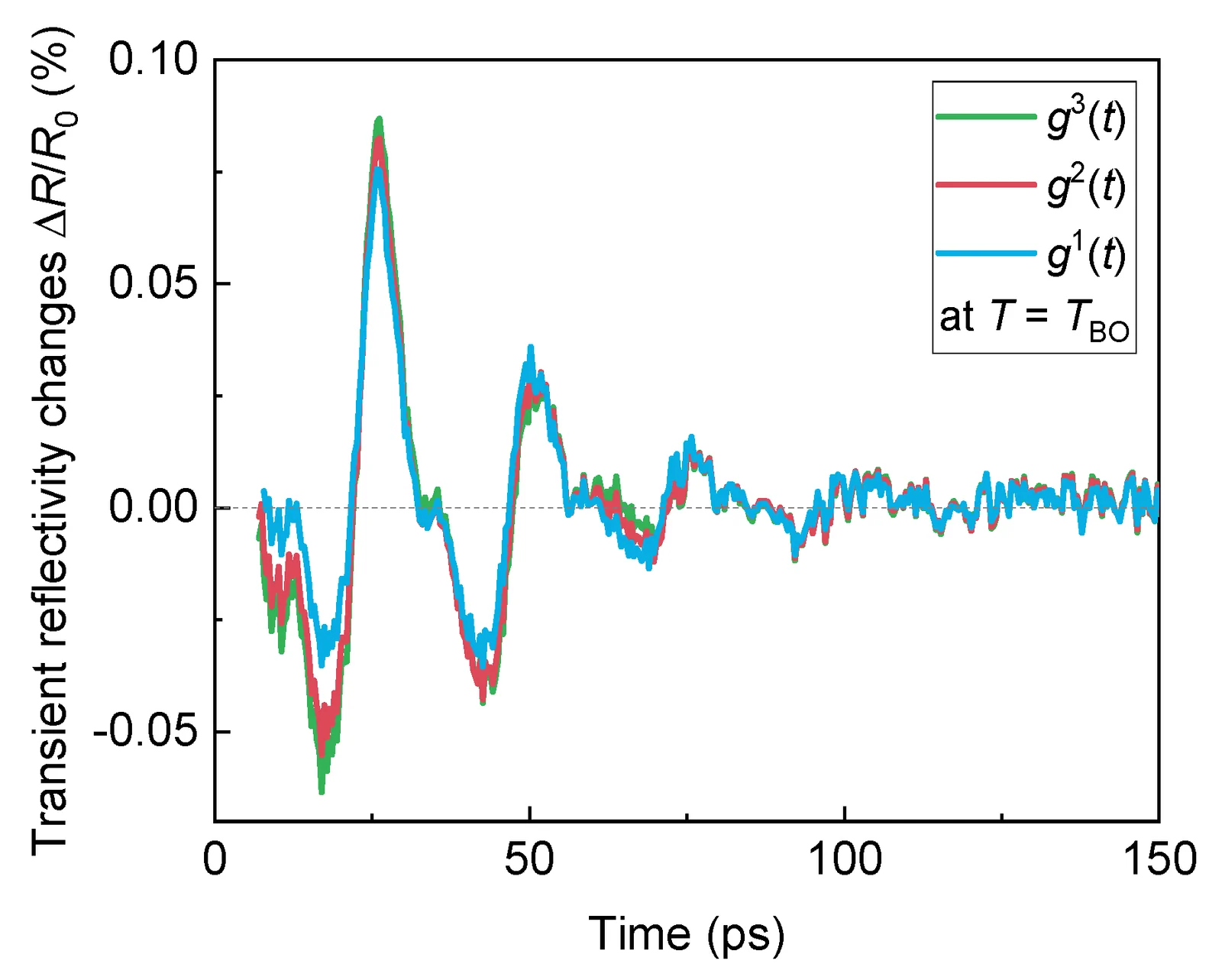
Transient reflectivity changes Δ*R*(*t*)/*R*_0_, caused by reverberating acoustic pulses in the film, were reconstructed from the experimental Δ*R*(*t*)/*R*_0_ signal at *F*_abs_ = 10.3 mJ/cm^2^ (Figure 1) using the time-domain functions *g*^1^(*t*), *g*^2^(*t*), and *g*^3^(*t*) at *T* = *T*_BO_, whose frequency responses are shown in Figure 6a,b.

As follows from Figure 7a, the discrepancy between the reconstructed Δ*R*(*t*)/*R*_0_ signals and the corresponding damped sine waves in the time interval Δ*t*_delay_ < 25 ps decreases with an increase in the filter order, as also demonstrated by the difference curve between the resulting output signals *g*^1^(*t*), *g*^2^(*t*), and *g*^3^(*t*), and the corresponding damped sine wave fits. Thus, applying higher-order filters results in a more accurate optical signal of propagating acoustic pulses in the substrate, referred to as Brillouin oscillations, due to a better attenuation of the optical signal from the thermal background and the reverberating acoustic pulses in the film. Again, by subtracting these improved Brillouin oscillations (*g*^2^(*t*) and *g*^3^(*t*) output signals) from the original experimental Δ*R*(*t*)/*R*_0_ signal, one can obtain the reconstructed transient reflectivity changes that are caused by reverberating acoustic pulses in the film. As seen from Figure 8, all the reconstructed echo-induced optical responses demonstrate nearly identical waveforms, with the maximum discrepancy occurring in the time interval Δ*t*_delay_ < 45 ps, which corresponds to the first echo. Increasing the filter order results in a slight increase in the amplitude of the first echo while generally preserving the main waveform features.

Now, one can make an attempt to isolate *f*_echo_ from *f*_BO_ instead, given that their central frequencies are significantly spaced apart. Figure 6c,d also show the frequency responses *H^n^*(*ω*) = sin^2n^(*ωT*/4) for *n* = 1, 3, 10 at *f*_echo_ = 39.1 GHz. In this case, for example, using *H*^1^(*ω*), *H*^3^(*ω*) and *H*^10^(*ω*) results in signal reduction at *f*_BO_ = 21.5 GHz to the much higher levels of 0.5766 (57.6%), 0.1917 (19.17%) and 0.0041 (0.41%), respectively. This is because the filter passband increases as the period *T* decreases. Figure 7b shows the results of isolating the Δ*R*(*t*)/*R*_0_ signal of reverberating acoustic pulses in the film (*f*_echo_) from the Δ*R*(*t*)/*R*_0_ signal of Brillouin oscillations in the substrate (*f*_BO_) for the experimental Δ*R*(*t*)/*R*_0_ signal at *F*_abs_ = 10.3 mJ/cm^2^ using time-domain functions *g*^1^(*t*), *g*^3^(*t*), and *g*^10^(*t*) at *T*_echo_ = 25.6 ps. The resulting output signal *g*^1^(*t*) at *T*_echo_ = 25.6 ps contains strong Brillouin oscillations of about 57.6% of their original amplitude and shows no apparent signs of reverberating acoustic pulses in the film. Meanwhile, the resulting output signal *g*^3^(*t*) at *T*_echo_ = 25.6 ps contains weaker Brillouin oscillations—about 19.17% of their original amplitude—and shows some periodic modulations of the waveform within the time interval of 100 ps that are different from the Brillouin oscillations. Finally, the resulting output signal *g*^10^(*t*) at *T*_echo_ = 25.6 ps clearly demonstrates the decaying periodic changes in the transient reflectivity signal, which are associated with the echoes in the film. Brillouin oscillations are now no longer visible, as they are strongly suppressed to a level of 0.41% of their original amplitude. However, it is worth noting that, by definition, the current technique is customized for isolating only sinusoidal signals (such as Brillouin oscillations). In our case, the 70 nm nickel film is relatively thick, so the optical response waveform of the acoustic pulses cannot be accurately modeled by a simple sinusoidal function [95]. Therefore, although technically the isolation of the Δ*R*(*t*)/*R*_0_ signal of echoes from the Δ*R*(*t*)/*R*_0_ signal of Brillouin oscillations is achievable, the reconstructed Δ*R*(*t*)/*R*_0_ signal of echoes in the nanoscale nickel film is not as accurate as the one reconstructed by the alternative method. This alternative approach isolates the Δ*R*(*t*)/*R*_0_ signal of Brillouin oscillations from the Δ*R*(*t*)/*R*_0_ signal of echoes and subsequently subtracts it from the original Δ*R*(*t*)/*R*_0_ signal, as shown in Figure 8. Nevertheless, it could be helpful for the retrieving of echo-induced pure sinusoidal signals when ultrathin optoacoustic transducer films of several nanometers in thickness are used [95].

**Figure 9:**
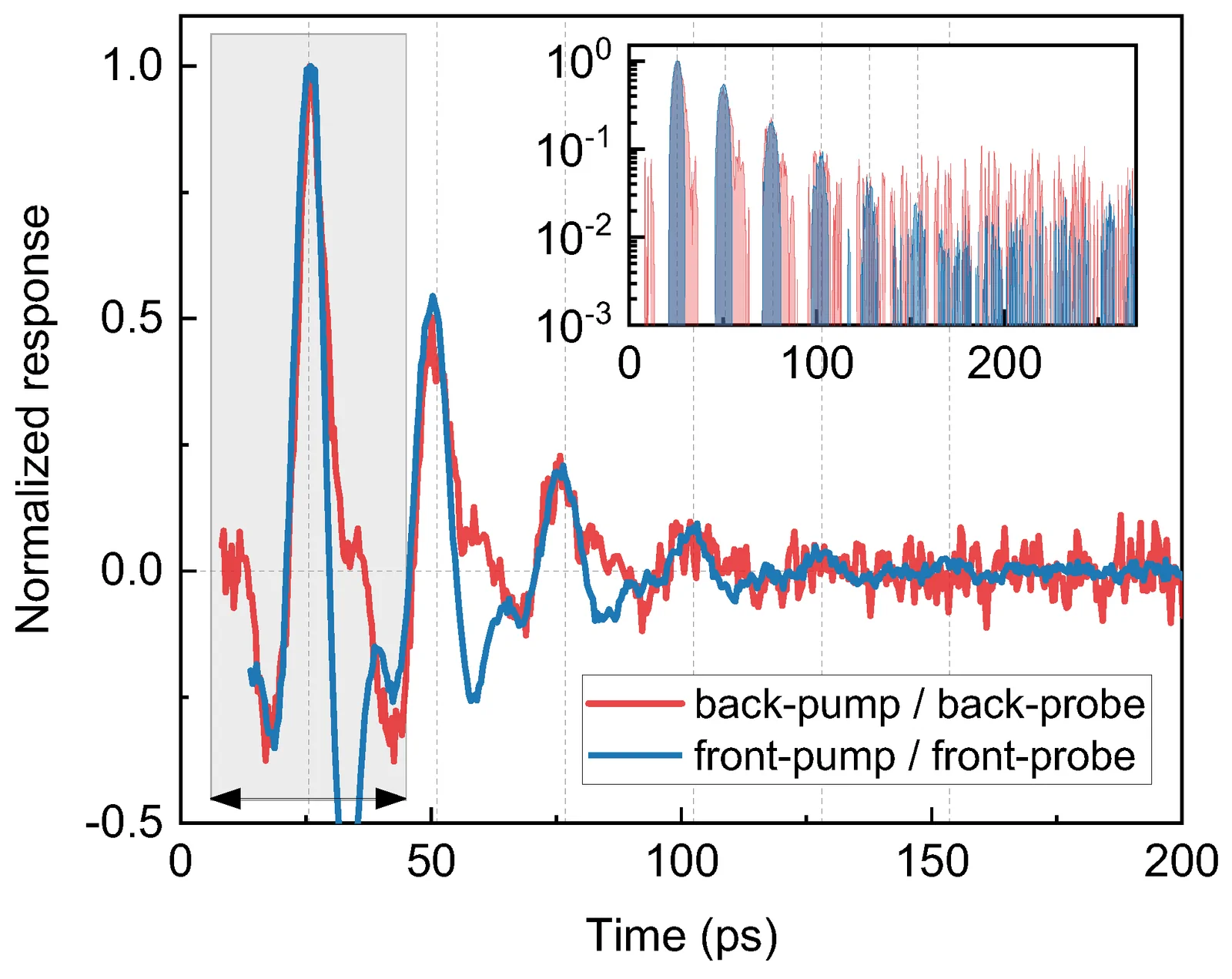
Comparison between the normalized Δ*R*(*t*)/*R*_0_ signals from acoustic echoes propagating back and forth within the nickel film as experimentally measured at the air–nickel interface (front-pump/front-probe configuration) and at the glass–nickel interface (back-pump/back-probe configuration). The vertical dashed lines denote the round-trip time of the reverberating acoustic pulses in the nanoscale nickel film. The inset shows the same graph with a logarithmic Y-axis.

Figure 9 compares the normalized Δ*R*(*t*)/*R*_0_ signals induced by reverberating acoustic pulses in the 70 nm thick nickel film, measured at the air–nickel interface (front-pump/front-probe configuration) and at the glass–nickel interface (back-pump/back-probe configuration).

**Figure A1:**
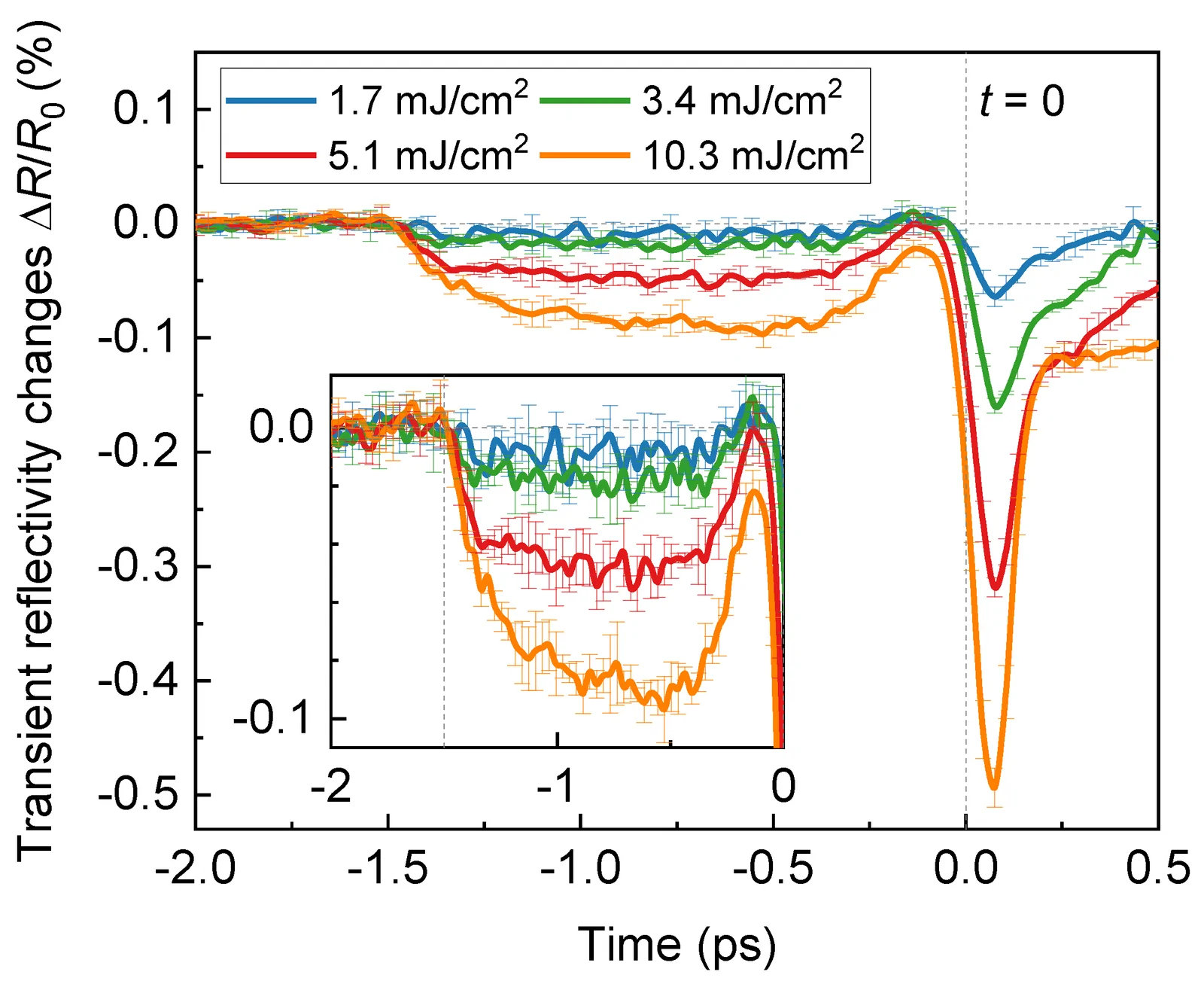
Transient reflectivity changes Δ*R*(*t*)/*R*_0_ (in %) of a borosilicate glass-supported, 70 nm thick nickel film in the back-pump/back-probe configuration (at the glass–nickel interface) over the pump-probe delay range (Δ*t*_delay_) from −2 to 0.5 ps with a time step of Δ*t*_1_ = 30 fs at absorbed pump fluences of *F*_abs_ = 1.7, 3.4, 5.1, and 10.3 mJ/cm^2^. At zero-time delay (Δ*t*_delay_ = 0), one can observe an ultrafast transient change in reflectivity in the negative domain (Δ*R*/*R*_0_ < 0), which comes after a less steep decrease in reflectivity that starts at Δ*t*_delay_ = −1.5 ps. The ultrafast transient at zero-time delay is not characteristic of nickel material at the probing wavelength of 793 nm immediately after excitation with a 3.1 eV femtosecond laser pulse at the air–nickel interface [80]. The decrease in reflectivity that starts at Δ*t*_delay_ = −1.5 ps is associated with the partial absorption of the pump pulse within the glass and the corresponding Δ*R*/*R*_0_ change detected in the glass: for a glass substrate with a thickness of 150 μm, the round-trip time of a light pulse in a medium with a refractive index *n* = 1.5 is *t* = 1.5 ps (2*d* = *ct*/*n,* where the speed of light *c =* 3 × 10^8^ m/s).

**Figure A2:**
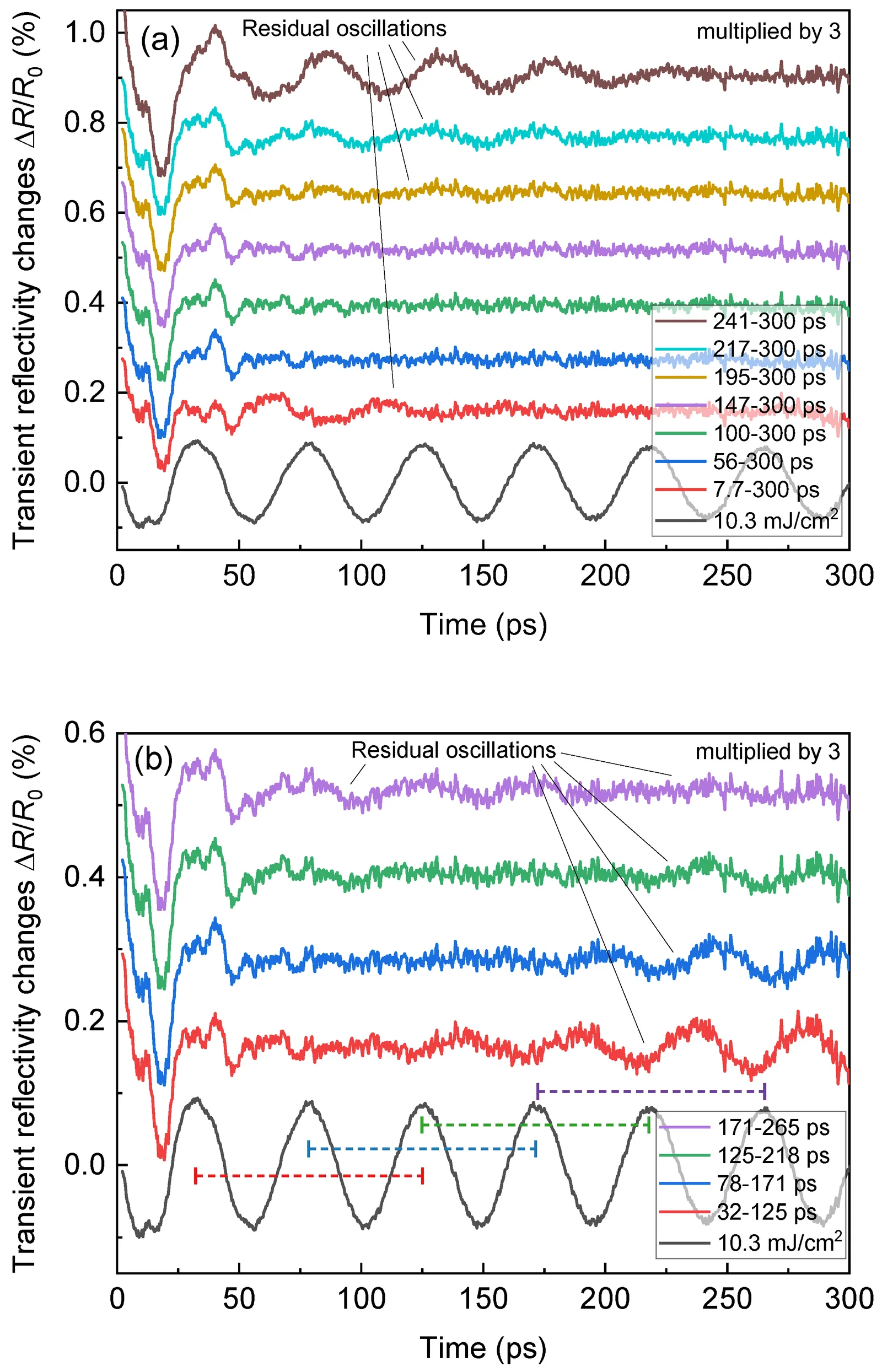
(**a**) For a glass-supported 70 nm thick nickel film: transient reflectivity changes Δ*R*(*t*)/*R*_0_ denote the residual difference between the Δ*R*(*t*)/*R*_0_ signal arising from acoustic pulses propagating in the substrate (i.e., Brillouin oscillations calculated via Expression (1) with *T*_BO_ = 46.6 ps) at *F*_abs_ = 10.3 mJ/cm^2^ and the damped sinusoidal fits (Expression (11)) over the cumulative time delay ranges: 7.7–300, 56–300, 100–300, 147–300, 195–300, 217–300, and 241–300 ps; (**b**) transient reflectivity changes Δ*R*(*t*)/*R*_0_ denoting the residual difference between the Δ*R*(*t*)/*R*_0_ signal from acoustic pulses propagating in the substrate (Brillouin oscillations using Expression (1) with *T*_BO_ = 46.6 ps) at *F*_abs_ = 10.3 mJ/cm^2^ and the damped sinusoidal fits (Expression (11)) for the sequential time delay ranges: 32–125, 78–171, 125–218, and 171–265 ps. Uncompensated residual Brillouin oscillations, resulting from the superposition of individual Brillouin oscillations with a constant frequency but a shifting phase, are observed. All curves (except for the original Brillouin oscillations at *F*_abs_ = 10.3 mJ/cm^2^) are multiplied by a factor of 3 for clarity.

**Figure A3:**
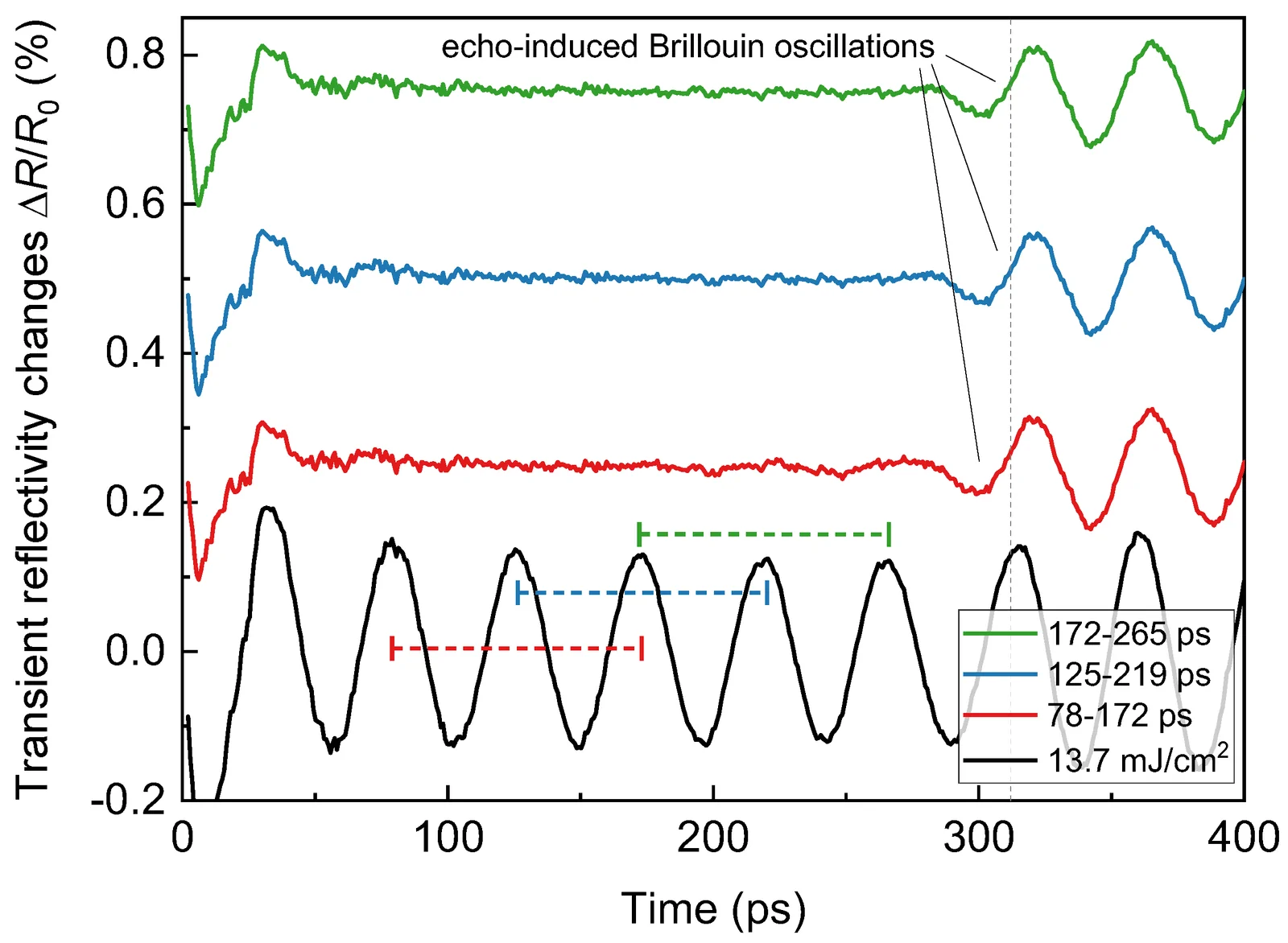
For 0.92 µm thick nickel film on glass: transient reflectivity changes Δ*R*(*t*)/*R*_0_ denoting the residual difference between the Δ*R*(*t*)/*R*_0_ signal from acoustic pulses propagating in the substrate (i.e., Brillouin oscillations calculated via Expression (1) with *T*_BO_ = 46.6 ps) at *F*_abs_ = 13.7 mJ/cm^2^ and the damped sinusoidal fits (Expression (11)) for the sequential time delay ranges: 78–172, 125–219, and 172–265 ps. No uncompensated residual Brillouin oscillations are observed, except for those induced by the echo (*T*_echo_ = 312 ps).

Both signal waveforms are highly correlated, particularly regarding the repetition period, the attenuation coefficient due to losses at the nickel–glass interface, and the rising edge of each acoustic echo. The discrepancy in the falling edge waveform can be attributed to the influence of the glass substrate. It should be noted that, normally, the duration of the acoustic pulse consisting of compression and tension regions and generated in the nickel film by a femtosecond laser pulse is around *τ*_ap_ ≈ 40 ps (as indicated by the double-sided arrow in Figure 9) as a result of thermal and electron diffusion within the film [16,90,92,96]. The acoustic pulse duration is estimated at the edges of the measured Δ*R*(t)/*R*_0_ signal, as previously established for nickel films with thicknesses of 120–200 nm [16,90,92,96]. In such films, the acoustic pulses do not overlap in time. However, since *T_echo_* < *τ*_ap_ for the 70 nm thick nickel film, characteristic distortions may be present in the waveform of the optical response within the region of acoustic pulse overlap. Furthermore, the detected optical signal can be distorted to some degree by the optical changes in the contacting glass substrate (see, for example, Figure A1 in Appendix A).

(2) To address the second problem, which is related to the effect of reverberating acoustic pulses leaking into the substrate on the amplitude-phase modulation of the resulting Brillouin scattering signal, the following approach is proposed. Here, to simulate the amplitude-phase modulation of Brillouin oscillations as a function of the number of acoustic pulses (*n*) leaking into the glass substrate from the metal film and their repetition period (*T*_echo_) at a constant wavelength of the probe radiation (λ_probe_), a simple but effective model can be used. The acoustic impedance of the film–substrate interface and the optoacoustic properties of the transparent substrate are assumed to be constant.

It should be recalled that the model considers a situation where a transparent sample (the glass substrate) is in contact with an absorbing material (the metal film). The pump light absorption at the film–substrate interface launches an acoustic pulse, which bounces back and forth within the film. Upon each interaction with the film–substrate interface, this acoustic pulse is partially transmitted into the substrate. The entire process is monitored with the time-delayed probe pulse focused through the substrate onto the film–substrate interface (as shown in Figure 1).

It is worth noting that in comparison to the theoretical framework in ultrafast photoacoustics, established by Thomsen et al. [15,16,17] and demonstrated recently, in particular, by Audoin [25], the current model does not describe, for example, the strain-induced reflectivity changes, which are periodically caused by the arrival of acoustic pulses reverberating within the film at the film–substrate interface [16]. The model proposed in this paper is designed to qualitatively describe the amplitude-phase modulation in the resulting waveform caused solely by the superposition of successive Brillouin oscillations (Figure 1). Strictly speaking, the theoretical framework [15,16,17,25] was established to describe how ultrashort light pulses generate and detect coherent longitudinal acoustic phonons at the surfaces of absorbing materials (metals) [16] and within transparent materials [15,17,25] in low excitation regimes. It assumes that laser-induced strain and optical reflectivity changes can be evaluated via a one-dimensional thermoelastic continuum framework with instantaneous thermalization and linear photoelastic coupling. However, at relatively high excitation fluences (close to the degradation or destruction threshold of the optoacoustic transducer film [32,80]), such as those used in the current paper, a highly non-equilibrium regime of the electronic and lattice subsystems occurs. Consequently, nonlinear phenomena in optoacoustic vibrations [93] should be taken into consideration, using, for example, two-temperature hydrodynamics, which integrates the two-temperature model with the compressible Navier–Stokes equations [97]. In the latter case, the situation is exacerbated by the lack of experimental validation for the numerous simulations predicting the behavior of the electron–phonon coupling parameter, electron thermal conductivity, and electron heat capacity over a wide range of electron temperatures from several hundred to several thousand degrees [98,99].

In principle, the model implies that each acoustic pulse leaking from the film into the substrate causes, upon interacting with the probe light, its own individual Brillouin oscillations (abbreviated hereinafter as *IBO_n_*, where *n* is the index number). These oscillations share the same frequency but differ in phase, thereby representing phase-shifted waveforms of an identical frequency. Now, as was previously shown in Expression (11), an *IBO_n_* can be described as an exponentially damped sinusoidal waveform with a slight variation in the added phase shift *ψ* and a smoothing function:

(14) $$\mathrm{IB}O_{n}(t)=A_{n}\times \exp\left(-\frac{t+\psi_{n}}{t_{0}}\right)\times \sin\left[\frac{2\pi }{T}(t-\varphi +\psi_{n})\right]\times \frac{a}{1+\exp\left[-b\left(t-\psi_{n}\right)\right]},$$

where *A_n_* is the amplitude, *t*_0_ is the damping time constant*, T* is the period, *φ* is the initial phase shift, and *ψ_n_* is the additional phase shift in the *n*-th Brillouin oscillation waveform that is caused by each time-delayed acoustic pulse and determined by the value of *T*_echo_. Furthermore, *t* is the time coordinate, while *a* and *b* are smoothing coefficients. For the *n*-th waveform of Brillouin oscillations (*n* ≥ 1) the additional phase shift and amplitude are described by *ψ_n_* = *T_echo_*(*n* − 1) and $A_{n}=t_{\mathrm{fs}}^{n-1}A_{1}$, respectively. Here, *T*_echo_ is the round-trip time of an echo in the film*, t_fs_* is the transmission coefficient of acoustic pulses through the film–substrate interface, and *A*_1_ is the amplitude of Brillouin oscillations generated by the initial acoustic pulse. Note that the exponential decay must be delayed by *t* = *T*_echo_ for each subsequent waveform of Brillouin oscillations. This implies that the *n*-th exponential decay equals to exp*_n_* = exp(−(*t* + *ψ_n_*)/*t*_0_) only for *t* ≥ *ψ_n_*, otherwise, it equals exp*_n_* = 1. In addition, a sigmoidal logistic function *a*/(1 + exp[−*b*(*t* − *ψ_n_*)]) with *a* = 1 and *b* = 0.3 1/ps can be used to ensure the smoothness of the leading edge of the simulated sinusoidal waves, replacing their abrupt appearance at times *t* = *ψ_n_* (*n* ≥ 1). Thus, for the initial acoustic pulse (*n* = 1), the additional phase shift is *ψ*_1_ = 0 ps, the amplitude is *A*_1_, the exponential decay is exp(−*t*/*t*_0_), and the sigmoidal logistic function is *a*/(1 + exp[−*bt*]).

The resulting Brillouin oscillations (*RBO*), detected in the transparent substrate at time delays *t* > *T*_echo_ in the back-pump/back-probe configuration (Figure 1), are assumed to be the sum of these individual Brillouin oscillations:

*RBO*(*t*) = *IBO*_1_(*t*) + *IBO*_2_(*t*) + *IBO*_3_(*t*) + … (15)

Therefore, the resulting Brillouin oscillation signal is modeled as a linear sum of the individual oscillations created by each separate acoustic pulse. This linear superposition approach assumes that the individual acoustic waves do not strongly interfere with or change each other as they propagate and interact in the medium.

Now, to apply the above-mentioned Expressions (14) and (15) and simulate the superposition of Brillouin oscillations for the current experimental conditions, one must know the exact values of the initial phase shift (*φ*), damping time constant (*t*_0_), period of oscillations (*T*_BO_), transmission coefficient of the nickel–glass interface (*t_fs_*), and the additional phase shift (*ψ*). It is worth noting that since *T*_BO_ varies proportionally to the probing wavelength and inversely to the refractive index and sound velocity in the transparent substrate at normal incidence (*T*_BO_ = λ_probe_/(2*n*ν)), the simulation result is very sensitive to the probing wavelength.

**Figure 10:**
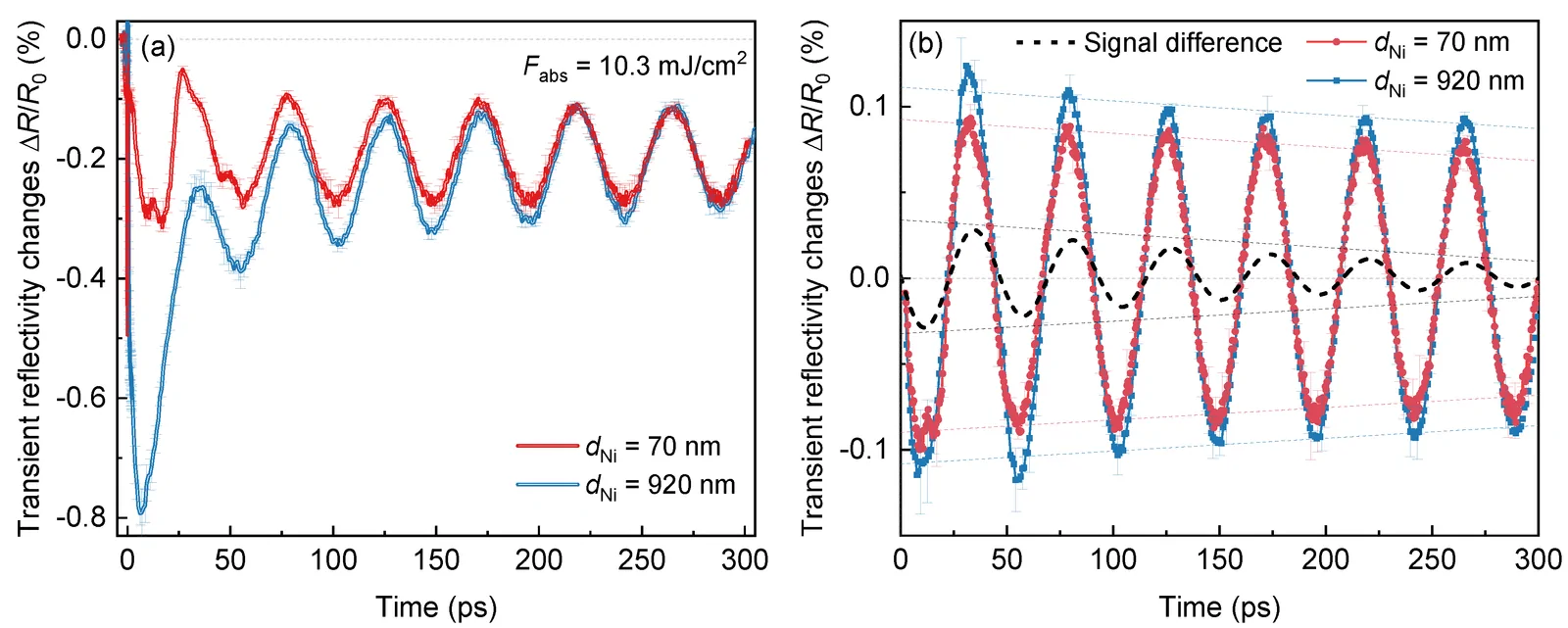
Transient reflectivity changes Δ*R*(*t*)/*R*_0_, measured in the back-pump/back-probe configuration at the same absorbed fluence of *F*_abs_ = 10.3 mJ/cm^2^, for the sample consisting of a glass substrate covered with a nickel optoacoustic transducer film of either 70 nm or 0.92 µm in thickness: (**a**) original transient reflectivity changes, Δ*R*(*t*)/*R*_0_; (**b**) reconstructed transient reflectivity changes Δ*R*(*t*)/*R*_0_ (Brillouin oscillations at *T*_BO_ = 46.6 ps), caused by propagating acoustic pulses in the glass substrate; the black dashed line is the difference signal between the Brillouin oscillations in the glass substrate covered with nickel films of different thicknesses.

First, the initial phase shift (*φ*), damping time constant (*t*_0_), and the period of Brillouin oscillations can be obtained from the experimental data. As is shown in Figure 3b and Figure 7a, approximating the reconstructed Brillouin oscillations for all the absorbed fluences in the range of 1.7 to 10.3 mJ/cm^2^ with an exponentially damped sine wave results in a period of Brillouin oscillations of *T*_BO_ = 46.6 ± 0.2 ps, a damping constant of *t*_0_ = 1.7 ± 0.1 ns, and an initial phase shift of *φ* = 20.3 ± 0.5 ps. Moreover, an experiment carried out in the same back-pump/back-probe configuration within the same range of absorbed fluences, but for a borosilicate glass substrate covered by a micrometer-thick nickel film, demonstrates a nearly identical initial phase shift value of *φ* = 21.0 ± 0.4 ps (see Figure 10). Thus, the initial phase shift *φ* of Brillouin oscillations experimentally detected in the same glass substrates, but covered with nickel films of various thicknesses (70 nm and 0.92 µm), proves to be nearly identical, averaging around 20.7 ± 0.5 ps.

Second, the acoustic impedance matching of the two contacting materials generally determines the reflection (*r*_fs_) and transmission (*t*_fs_) coefficients of the interface, where the subscripts *f* and *s* stand for the film and substrate, respectively. In addition, the interface quality (e.g., roughness, defects, impurities, and delamination) also strongly affects the transmission losses. Basically, as *t*_fs_ increases, the amplitude of the acoustic pulses leaking from the film into the substrate also rises, which in turn results in higher amplitudes of Brillouin oscillations from each successive echo in time-domain Brillouin scattering. Thus, higher *t*_fs_ values result in a stronger and more prolonged influence of each successive echo on the resulting waveform of Brillouin oscillations.

From the experimental data, it follows that the averaged reflection of acoustic pulses at the nickel–glass interface, for measurements from both air–nickel and nickel–glass interfaces, is *r*_exp_ = −0.43 ± 0.01 (Figure 9). As was mentioned earlier, the acoustic mismatch and diffuse mismatch models are widely used to model phonon transmission at interfaces. The acoustic mismatch model uses acoustic impedance to calculate reflection and transmission coefficients for phonons; it assumes that the contacting materials are continuous elastic media with perfect interfaces where phonon reflection and transmission are specular. In contrast, the diffuse mismatch model assumes that phonons scatter diffusely with probabilities determined by the phonon density of states and energy conservation. While both models can be used to approximate the experimental results, neither can accurately describe the full behavior within a wide range of temperatures or for different types of contacting materials. In particular, interfacial phonon modes, which play a key role in phonon transport across interfaces, are neglected in these models [6].

Here, as follows from the acoustic mismatch model [34], the acoustic impedances of the glass substrate and nickel film are *Z*_s_ = *v*_s·_*ρ*_s_ = 1.3 × 10^7^ kg/m^2^·s and *Z*_f_ = *v*_f·_*ρ*_f_ = 4.9 × 10^7^ kg/m^2^·s, respectively, where *v*_f_ = 5.47 nm/ps, *ρ*_f_ = 8900 kg/m^3^, *v*_s_ = 5.7 nm/ps, and *ρ*_s_ = 2230 kg/m^3^. The sound velocities in the metal film and glass substrate are calculated as *v*_f_ = 2*d*/*T*_echo_ = 5.47 nm/ps and *v*_s_ = λ_probe_/(2*nT*_BO_) = 5.67 nm/ps (*T*_echo_ = 25.6 ps, *d* = 70 nm, *n* = 1.5 for the glass substrate at *λ*_probe_ = 793 nm, *T*_BO_ = 46.6 ps). Thus, the amplitude reflection and transmission coefficients of acoustic pulses at the nickel–glass interface at normal incidence are *r*_fs_ = (*Z*_s_ − *Z*_f_)/(*Z*_s_ + *Z*_f_) = −0.59 and *t*_fs_ = 2*Z*_s_/(*Z*_s_ + *Z*_f_)) = 0.41. Consequently, *r*_exp_ < *r*_fs_. If *r*_exp_ > *r*_fs_, it would indicate the delamination of the film from the substrate. On the contrary, the condition *r*_exp_ < *r*_fs_ indicates imperfect air–nickel and nickel–substrate interfaces, as the increased roughness leads to diffuse phonon scattering. The arithmetic mean roughness (*R*_a_) of the free surface of the nickel film over an area of 10 × 10 μm^2^ was measured using atomic force microscopy to be of 6 nm. Additionally, the presence of an oxide film on top of the nickel film can affect the impedance ratio at the air–nickel interface. It is also possible that the absorption of the propagating acoustic pulse by the nickel film itself, which is heated to hundreds of degrees Kelvins at such high absorbed laser fluences, may increase [32]. In addition, while the sound velocities in the nickel film and the glass substrate were taken from these experimental measurements, the densities were obtained from the reference data for the respective bulk (macroscopic) materials. However, as the density of a deposited film is generally lower than that of its bulk counterpart (i.e., ρ_film_ ≤ ρ_bulk_), the real acoustic impedance of the nickel film is also most likely lower than the calculated value [100]. Thus, it is not possible to unambiguously estimate the transmission of the nickel–glass interface from the experimental data alone. Therefore, the transmission coefficient *t*_fs_ = 0.41 predicted by the acoustic mismatch model was used in the simulation. Consequently, the transmission coefficient of the nickel–glass interface significantly contributes to the amplitude-phase modulation of the resulting Brillouin oscillation waveform.

**Figure 11:**
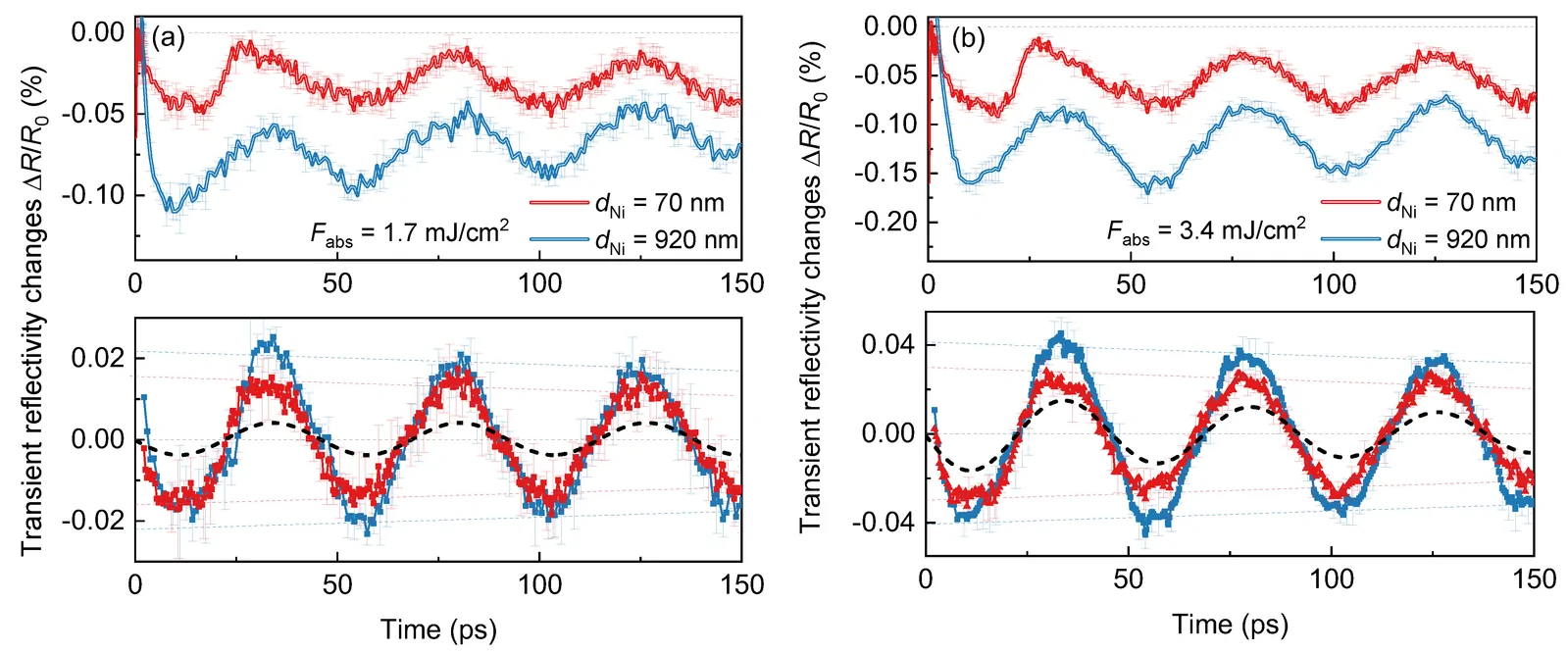
(**upper** panels) Transient reflectivity changes Δ*R*(*t*)/*R*_0_ measured in the back-pump/back-probe configuration at absorbed fluences of *F*_abs_ = 1.7 (**a**) and 3.4 (**b**) mJ/cm^2^ for a sample consisting of a glass substrate covered with a nickel optoacoustic transducer film with thicknesses of either 70 nm or 0.92 µm; (**lower** panels) reconstructed transient reflectivity changes Δ*R*(*t*)/*R*_0_ (Brillouin oscillations at *T*_BO_ = 46.6 ps) caused by propagating acoustic pulses in the glass substrate: (**a**) original and reconstructed transient reflectivity changes Δ*R*(*t*)/*R*_0_ at the same absorbed fluence of *F*_abs_ = 1.7 mJ/cm^2^; (**b**) original and reconstructed transient reflectivity changes Δ*R*(*t*)/*R*_0_ at the same absorbed fluence of *F*_abs_ = 3.4 mJ/cm^2^. The black dashed line represents the difference signal between the Brillouin oscillations in the glass covered with nickel films of different thicknesses.

Now, before proceeding to the modeling, it is worth noting that depending on the film thickness of the optoacoustic transducer, and at the same absorbed laser fluence, the resulting amplitude of Brillouin oscillations in the glass substrate was found to vary. For example, as seen in Figure 11b, at the same absorbed fluence of *F*_abs_ = 10.3 mJ/cm^2^ over a time range of 300 ps, the resulting amplitude of Brillouin oscillations for the nanoscale-thick (70 nm) nickel film is approximately 20% lower than that for the much thicker submicron-thick (0.92 µm) nickel film. Furthermore, as shown in Figure 11a,b, a similar pattern is also observed for lower absorbed laser fluences, such as *F*_abs_ = 1.7 and 3.4 mJ/cm^2^; in these cases, the resulting amplitude of Brillouin oscillations for the 70 nm nickel film is around 25% and 30% lower than that for the 0.92 µm nickel film. As will be shown below, this discrepancy in the amplitude of Brillouin oscillations as a function of the thickness of the optoacoustic transducer film is attributed to the specific superposition of individual waveforms of Brillouin oscillations from every acoustic pulse leaking into the substrate from the metal film.

**Figure 12:**
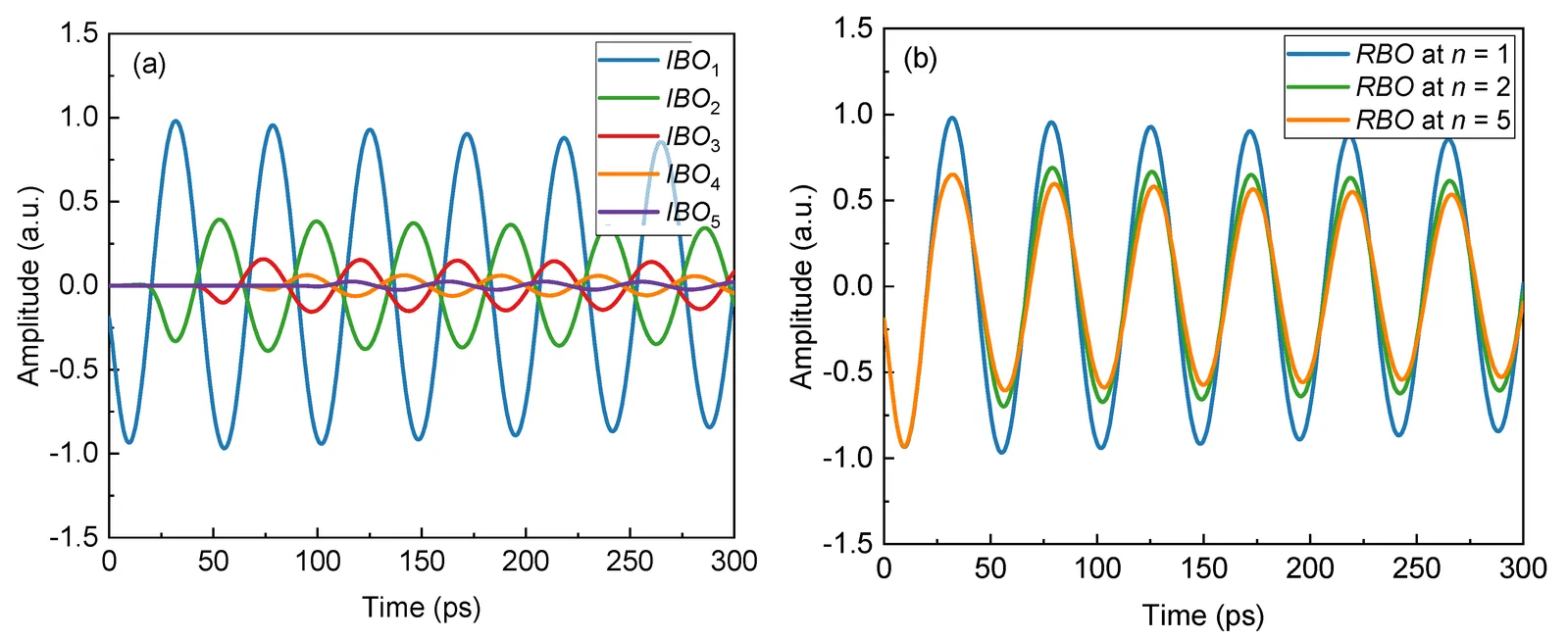
(**a**) Simulation of individual Brillouin oscillation (*IBO*_n_, *n* = 1, 2, 3, 4, and 5) waveforms induced by the respective acoustic pulses leaking from the film into the substrate; (**b**) simulation of the resulting Brillouin oscillation waveform due to the superposition of the *n* = 2 and *n* = 5 individual Brillouin oscillation waveforms in comparison with the unmodulated Brillouin oscillation waveform (*n* = 1).

Now, in Figure 12 one can see the simulation results of individual waveforms of Brillouin oscillations (*IBO*_n_) and their superposition (*RBO*) calculated using Expressions (14) and (15) for the glass substrate, covered with a 70 nm nickel film. Figure 12a shows the individual waveforms of Brillouin oscillations (*IBO*_n_) induced by each acoustic pulse (*n* = 1, 2, 3, 4, and 5) leaking into the substrate from the film with a round-trip time of *T*_echo_ = 25.6 ps; the period of Brillouin oscillations of *T*_BO_ = 46.6 ps; the initial phase shift in *φ* = 20.37 ps; and the transmission coefficient of *t*_fs_ = 0.41. One can see that the individual waveforms of Brillouin oscillations are almost out of phase. Therefore, their superposition results in a decrease in the resulting amplitude of Brillouin oscillations and a slight phase shift. Figure 12b shows the resulting waveforms of Brillouin oscillations due to the superposition of *n* = 2 and *n* = 5 individual Brillouin oscillations in comparison with the unmodulated waveform of Brillouin oscillations induced by a probe light scattering off a single acoustic pulse (*n* = 1) over a time range of 300 ps.

In fact, this is equivalent to comparing the resulting waveforms of Brillouin oscillations in the glass substrate covered with the nickel films of either 70 nm (*T*_echo_ = 25.6 ps) or 0.92 µm (*T*_echo_ *= 2d/v_f_ >* 300 ps) in thickness. As the modeling shows, for the nanoscale 70 mm thick nickel film, the resulting amplitude of Brillouin oscillations is around 38% lower than the amplitude obtained with the 0.92 µm thick nickel film. Experimentally, as shown in Figure 10 and Figure 11, this discrepancy is approximately 25% on average. This suggests that the actual transmission coefficient (*t*_fs_) of the nickel–glass interface differs from that predicted by the acoustic mismatch model. In addition, the thicknesses of the nickel films differ by more than one order of magnitude, which may result in different crystallographic forms that affect the attenuation of reverberating high-frequency phonons.

As clearly shown in Figure 12a,b, the experimental results do not reveal any observable effect of acoustic pulses leaking from the nanoscale (70 nm) nickel film into the substrate on the resulting amplitude of Brillouin oscillations. This absence is due to the fact that the amplitude modulation initiates at times *t* > *T*_echo_ = 25.6 ps, which is nearly half the period of the Brillouin oscillations (*T*_BO_ = 46.6 ps), i.e., *T*_echo_ ≈ 0.55*T*_BO_.

**Figure 13:**
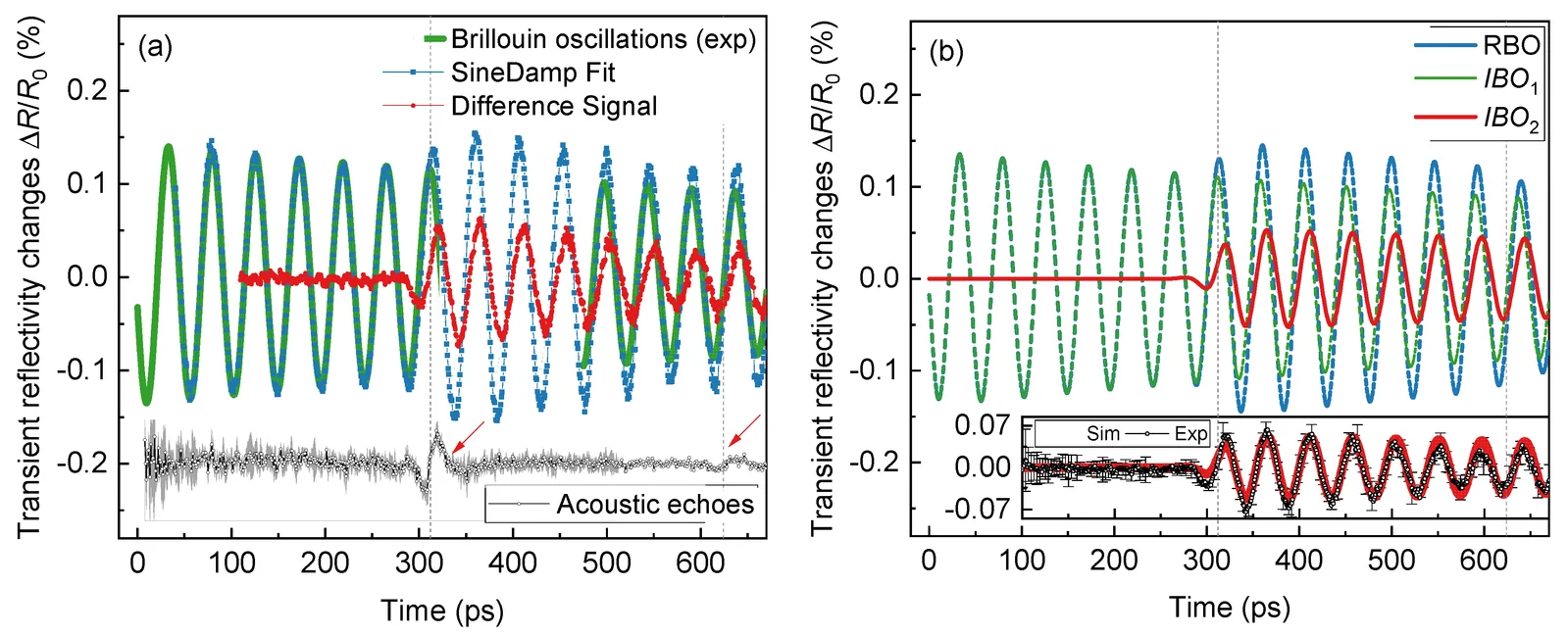
(**a**) Experimentally measured Brillouin oscillations in glass covered by a 0.92 µm thick nickel film at the absorbed fluence of *F*_abs_ *=* 13.7 mJ/cm^2^ in the time interval up to 670 ps (blue line), approximated with an exponentially decaying sine wave in the 50–280 ps range (green line); their difference signal reveals secondary Brillouin oscillations induced by an echo pulse (red line); below is an echo-induced signal with an offset measured at the air–nickel interface; (**b**) simulation of individual Brillouin oscillations (*IBO*_n_, *n* = 1 and 2) waveforms induced by the initial acoustic pulse and its echo leaking from the film into the substrate, and their superposition as the resulting Brillouin oscillation (*RBO*) waveform; the inset shows a comparison between the experimental and simulated individual waveforms of Brillouin oscillations.

Instead, the modulation of the resulting waveform of Brillouin oscillations can be clearly seen when a much thicker film, such as the 0.92 µm thick nickel film, is used as an optoacoustic transducer. In this case, the round-trip time is *T*_echo_ = 312 ps which results in *T*_echo_ ≈ 6.7*T*_BO_. Figure 13a,b show, respectively, the experimentally measured and simulated time-domain Brillouin scattering signals in the glass substrate covered with the 0.92 µm thick nickel film within a time delay range up to *t* = 670 ps > 2*T*_echo_ at an absorbed fluence *F*_abs_ *=* 13.7 mJ/cm^2^. As seen from Figure 13a, the reconstructed waveform of the resulting Brillouin oscillations detected in the experiment clearly shows an amplitude-phase modulation starting from a time delay around 300 ps, which corresponds to the round-trip time in the film (*T*_echo_ = 312 ps). The Δ*R*(*t*)/*R*_0_ signal of acoustic echoes reverberating within the film, which was additionally measured at the air–film interface at nearly the same absorbed fluence of *F*_abs_ *=* 9.9 mJ/cm^2^, is also shown in Figure 13a (two echoes leaking from the film into the substrate at 312 and 624 ps are visible and marked with red arrows). As seen in Figure 13a, the experimental waveform of Brillouin oscillations shows an exponential damping from the beginning until around 300 ps. After the acoustic echo leaks from the film into the substrate at 312 ps, the experimental waveform of the Brillouin oscillations shows an increase in the amplitude back to its initial value and a phase shift of several picoseconds. This behavior is clearly visible after first approximating the time interval from 50 to 280 ps (at times *t* < *T*_echo_) with an exponentially decaying sinusoidal wave (Expression (11)) using the parameters *φ* = 21.0 ps, *T =* 46.46 ps, *t*_0_ = 1.4 ns, and *A* = 1.4 × 10^−3^, and, subsequently subtracting this approximation from the experimental signal. The resulting difference signal reveals a second waveform of Brillouin oscillations starting at around 290 ps. This secondary waveform has the same frequency and a lower amplitude but is phase-shifted; it is related to the Brillouin scattering of the probe light by the acoustic echo pulse leaking from the film into the substrate at around 312 ps. Figure 13b, respectively, shows the results of the modeling of the amplitude-phase modulation of the resulting waveform of Brillouin oscillations; Expressions (14) and (15) were used with parameters *T*_echo_ *=* 312 ps, *T*_BO_ *=* 46.46 ps, *t*_0_ = 1.4 ns, *t*_fs_ = 0.41, *a* = 1, and *k* = 0.1 ps^−1^. One can see the individual Brillouin oscillations—*IBO*_1_ and *IBO*_2_—induced respectively by the initial acoustic pulse and the subsequent echo pulse, as well as their superposition forming the resulting Brillouin oscillations (*RBO*) detected in the experiment. The inset in Figure 13b compares the experimental and simulated individual waveforms of Brillouin oscillations induced by probe light scattering on the acoustic echo pulse leaking from the film into the substrate at around 312 ps.

Thus, the experimental and simulation data are consistent with each other. They demonstrate that the amplitude of the resulting Brillouin oscillation waveform within the transparent substrate could be manipulated by adjusting the phase shifts of individual Brillouin oscillation waveforms through changing, for example, the thickness of the optoacoustic transducer’s metallic film.

It is also shown that, unlike the sufficiently thick submicron nickel film (*d* = 0.92 µm), which does not exhibit amplitude-phase modulation by reverberating acoustic pulses during the first several hundred picoseconds (as *T*_echo_ >> *T*_BO_, *T*_echo_ = 312 ps, *T*_BO_ = 46.6 ps in glass for *λ*_probe_ = 793 nm), the nanometer-scale (*d* = 70 nm) nickel film—where *T*_echo_ ≈ *T*_BO_/2 (*T*_echo_ = 25.6 ps)—causes a significant reduction (25% on average) in the amplitude of the resulting Brillouin oscillations within the transparent substrate, reaching nearly its minimum due to destructive interference (as shown in Figure 12b).

At the same time, it has been demonstrated for the submicron nickel film (*d* = 0.92 µm) that the amplitude of the resulting Brillouin oscillations increases due to the constructive interference of individual waveforms of Brillouin oscillation at times *t* > *T*_echo_. Thus, the maximum increase in the amplitude of the resulting Brillouin oscillations is expected to occur when using films that satisfy the condition *T*_echo_ ≈ 2^n−1^*T*_BO_ (where *n* is a natural number, *n* ≥ 1). In glass, at a probe wavelength of *λ*_probe_ = 793 nm (*T*_BO_ = 46.6 ps) this requires using relatively thick optoacoustic transducer nickel films with thicknesses *d* = 0.5 *v*_f_ *T*_echo_ = 2^n−2^ *v*_f_ *T*_BO_, for example, this corresponds to *d* = 115–140 nm at *n* = 1 and *d* = 920–1120 nm at *n* = 4 (assuming a sound velocity in the range of 5–6 nm/ps). However, this approach may not be practical, as the increase in the resulting amplitude is delayed by *t* = 2^n−1^ *T*_BO_ (as shown in Figure 13 for the thick submicron film). To obtain an initial increase in the resulting amplitude of Brillouin oscillations, it is necessary to use nanoscale films where *T*_echo_ < *T*_BO_/4. For nanoscale films where *T*_echo_ ≈ *T*_BO_/4 (*d* = 29–35 nm for sound velocities ranging from 5 to 6 nm/ps), the amplitude modulation of the resulting Brillouin oscillations is minimized because the individual Brillouin oscillations are phase-shifted by 45°. Of course, from an experimental point of view, these values are only rough estimates, as the sound velocity in the film strongly depends on the deposition parameters. Notably, the increase or decrease in the resulting amplitude is considered relative to the unmodulated amplitude (i.e., in the absence of acoustic echoes, such as shown in the time interval *t* < *T*_echo_ = 312 ps for the submicron film in Figure 13).

It also should be noted that intrinsic acoustic attenuation within the nickel film is not considered in the current model. On the one hand, it has been shown that intrinsic acoustic attenuation within nickel films with thicknesses ranging from several hundred nanometers to several micrometers can be safely neglected [90,101] due to the relatively low value of the ultrasonic attenuation measured in the 9–100 GHz range. On the other hand, it has been shown that acoustic attenuation cannot be neglected for nickel films of several hundred nanometers in thickness due to the ultra-high frequency involved in picosecond acoustics: due to frequency-dependent attenuation, the acoustic pulse initially suffers a loss of its highest frequency components, which consequently leads to a broadening of the echo and a lowering of its amplitude [53]. As a result, it affects the absolute value of the reflection/transmission coefficient [53]. In our case, for the 70 nm nickel film no broadening of the echoes was observed and the measured averaged reflection coefficient of acoustic pulses in the nickel–glass interface (Figure 9) with that predicted by the acoustic mismatch theory. However, one should acknowledge that the duration of the acoustic pulses measured in the 0.92 μm nickel film (the inset in Figure 13a) is greater than that in the 70 nm nickel film (Figure 9), which could also be due to a different crystal structure. Therefore, while neglecting the acoustic attenuation can indeed in some cases result in the overestimation of the reflection coefficient in the experiment [53], which affects the amplitude of the echo-induced Brillouin oscillations, several studies have shown that for nickel films such a parameter can be safely neglected [90,101]. It is worth noting, however, that care should be taken when preparing films of significantly different thicknesses to ensure that their crystal structure is preserved as much as possible, provided that attenuation is not a critical parameter when comparing the samples.

Ultimately, it is experimentally and theoretically demonstrated in this study that studying transparent materials using time-domain Brillouin scattering requires taking into account such an important factor as the effect of reverberating acoustic pulses in the optoacoustic transducer film on the resulting (detected) waveform of Brillouin oscillations. It is of particular importance when the attenuation of high-frequency phonons is studied by measuring the damping constant of Brillouin oscillations [12,17,61], or when any modulation of the detected Brillouin oscillations is related to the structural or compositional changes within the material under study [23,55]. In the back-pump/back-probe configuration to prevent the amplitude-phase modulation of the resulting Brillouin oscillations, one can alternatively use sufficiently thick films of optoacoustic transducers so as to enable the investigation of the desired depth of the material with a single acoustic pulse. Furthermore, in accordance with the acoustic mismatch model, matching the acoustic impedance of a thin film to that of a substrate under study maximizes phonon transmission across the interface and minimizes acoustic reflection, which is beneficial in this case.

## 5. Conclusions

The effects accompanying the nanoscale characterization of thin-film metal–dielectric structures through the substrate—using ultrafast laser pulses to generate and detect high-amplitude, high-frequency acoustic phonons—are experimentally and theoretically studied. For a glass-supported 70 nm nickel polycrystalline film, measurements of the transient reflectivity changes Δ*R*(*t*)/*R*_0_ at 793 nm (1.56 eV) were carried out by focusing femtosecond pump and probe laser pulses through the substrate at the glass–nickel interface (in the back-pump/back-probe configuration) at absorbed laser fluences ranging from 1.7 to 10.3 mJ/cm^2^, which generated peak pressures of up to several gigapascals. The measured Δ*R*(*t*)/*R*_0_ signal was dominated by the resulting high-amplitude Brillouin oscillations within the substrate at a frequency of *f*_BO_ = 1/*T*_BO_ = 21.5 ± 0.1 GHz.

Firstly, within the range of absorbed fluences from 1.7 to 10.3 mJ/cm^2^, the transient reflectivity signal—induced by the reverberation of acoustic pulses in the film (echoes) and obscured by high-amplitude Brillouin oscillations within the substrate—was reconstructed at a frequency of *f*_echo_ = 1/*T*_echo_ = 39.1 ± 0.2 GHz. This reconstruction was performed via post-processing by applying a zero-phase, high-pass, finite impulse response (FIR) filter in the time domain. This filter demonstrates unconditional stability and computational simplicity, while offering adjustable parameters for central frequency and passband selection. The reconstructed, echo-induced optical response waveform was found to agree with the directly measured echo-induced signal at the air–nickel interface in the front-pump/front-probe configuration.

Secondly, the effect of these acoustic echoes leaking from the film into the substrate on the amplitude-phase modulation of the resulting waveform of Brillouin oscillations was experimentally and theoretically studied. To demonstrate this, nanoscale and submicroscale nickel films acting as the optoacoustic transducer were used. The detected resulting Brillouin oscillations were found to undergo amplitude-phase modulation due to the superposition of the individual Brillouin oscillations generated by the acoustic pulses leaking into the substrate after reverberation in the film. It is shown that, unlike sufficiently thick submicron films that exhibit no amplitude-phase modulation by reverberating acoustic pulses during the first several hundred picoseconds (*T*_echo_ >> *T*_BO_), nanoscale films (where *T*_echo_ ≈ *T*_BO_/2) cause a significant reduction in the resulting amplitude of the Brillouin oscillations in the substrate. This amplitude reaches its minimum due to destructive interference.

The reconstructed transient reflectivity changes Δ*R*(*t*)/*R*_0_ induced by reverberating acoustic pulses within the film and by propagating acoustic pulses within the substrate were analyzed within the entire fluence range. The dependencies of the transient reflectivity changes Δ*R*(*t*)/*R*_0_, which represent the amplitude of the Brillouin oscillations and the amplitude of the first acoustic echo on the absorbed laser fluence, were found to exhibit linear scaling from the origin. Thus, within the range of relatively high but non-destructive absorbed fluences from 1.7 to 10.3 mJ/cm^2^, the peak pressures in laser-induced compression and tension regions of propagating acoustic pulses were estimated to be in the range from 1.1 to 6.5 GPa in the nanoscale nickel film and from 0.2 to 1.2 GPa in the borosilicate glass substrate. The maximum absorbed fluence of 10.3 mJ/cm^2^ at a repetition rate of 0.5 kHz was determined to be the upper threshold for non-destructive testing of the glass-supported 70 nm nickel film. A further increase in fluence was found to result in the degradation and destruction of the nanoscale nickel film of optoacoustic transducer.

Controlling the amplitude of Brillouin oscillations in a transparent material through varying the optoacoustic transducer film thickness was demonstrated. Optimal thicknesses for increasing and decreasing the amplitude of Brillouin oscillations were proposed based on the experimental results and theoretical simulations. Investigating picosecond transient processes in condensed matter under extreme thermomechanical loads—induced by ultrashort laser pulses near the damage threshold—is crucial for validating, improving and developing theoretical models. This research also supports the ongoing downscaling of fundamental microelectronic elements, which leads to increased specific heat generation and intensified localized thermal dissipation challenges.

## Data Availability

The original contributions presented in this study are included in the article. Further inquiries can be directed to the corresponding author.

## Abbreviations

The following abbreviations are used in this manuscript:

BO	Brillouin oscillations
IBO	Individual Brillouin oscillations
RBO	Resulting Brillouin oscillations
FIR	Finite impulse response
